# Utilization of Reimbursed Acupuncture Therapy for Low Back Pain

**DOI:** 10.1001/jamanetworkopen.2024.30906

**Published:** 2024-08-29

**Authors:** Molly Candon, Arya Nielsen, Jeffery A. Dusek, Sebastian Spataro Solorzano, Martin Cheatle, Mark D. Neuman, Craig Samitt, Siyuan Shen, Rachel M. Werner, David Mandell

**Affiliations:** 1 Department of Psychiatry, Perelman School of Medicine, University of Pennsylvania, Philadelphia; 2 Department of Health Care Management, Wharton School, University of Pennsylvania, Philadelphia; 3 Leonard Davis Institute of Health Economics, University of Pennsylvania, Philadelphia; 4 Department of Family Medicine & Community Health, Icahn School of Medicine at Mount Sinai, New York, New York; 5 Susan Samueli Integrative Health Institute, University of California, Irvine; 6 Division of General Internal Medicine, Department of Medicine, University of California, Irvine; 7 Rice University, Houston, Texas; 8 Department of Anesthesiology and Critical Care, Perelman School of Medicine, University of Pennsylvania, Philadelphia; 9 Department of Medicine, Perelman School of Medicine, University of Pennsylvania, Philadelphia; 10 ITO Advisors, Paradise Valley, Arizona

## Abstract

**Importance:**

Treating low back pain (LBP) often involves a combination of pharmacologic, nonpharmacologic, and interventional treatments; one approach is acupuncture therapy, which is safe, effective, and cost-effective. How acupuncture is used within pain care regimens for LBP has not been widely studied.

**Objective:**

To document trends in reimbursed acupuncture between 2010 and 2019 among a large sample of patients with LBP, focusing on demographic, socioeconomic, and clinical characteristics associated with acupuncture use and the nonpharmacologic, pharmacologic, and interventional treatments used by patients who utilize acupuncture.

**Design, Setting, and Participants:**

This cross-sectional study included insurance claims of US adults in a deidentified database. The study sample included patients diagnosed with LBP between 2010 and 2019. Data were analyzed between September 2023 and June 2024.

**Main Outcomes and Measures:**

Changes in rates of reimbursed acupuncture utilization between 2010 and 2019, including electroacupuncture use, which involves the electrical stimulation of acupuncture needles. Covariates included age, sex, race and ethnicity, income, educational attainment, region, and a chronic LBP indicator. Secondary analyses tracked other nonpharmacologic treatments (eg, physical therapy, chiropractic care), pharmacologic treatments (eg, opioids, gabapentinoids), and interventional treatments (eg, epidural steroid injections).

**Results:**

The total sample included 6 840 497 adults with LBP (mean [SD] age, 54.6 [17.8] years; 3 916 766 female [57.3%]; 802 579 Hispanic [11.7%], 258 087 non-Hispanic Asian [3.8%], 804 975 non-Hispanic Black [11.8%], 4 974 856 non-Hispanic White [72.7%]). Overall, 106 485 (1.6%) had 1 or more acupuncture claim, while 61 503 (0.9%) had 1 or more electroacupuncture claim. The rate of acupuncture utilization increased consistently, from 0.9% in 2010 to 1.6% in 2019; electroacupuncture rates were relatively stable. Patients who were female (male: odds ratio [OR], 0.68; 99% CI, 0.67-0.70), Asian (OR, 3.26; 99% CI, 3.18-3.35), residing in the Pacific region (New England: OR, 0.26; 99% CI, 0.25-0.28), earning incomes of over $100 000 (incomes less than $40 000: OR, 0.59; 99% CI, 0.57-0.61), college educated (high school or less: OR, 0.32; 99% CI, 0.27-0.35), and with chronic LBP (OR, 2.39; 99% CI, 2.35-2.43) were more likely to utilize acupuncture. Acupuncture users were more likely to engage in other nonpharmacologic pain care like physical therapy (39.2%; 99% CI, 38.9%-39.5% vs 29.3%; 99% CI, 29.3%-29.3%) and less likely to utilize prescription drugs, including opioids (41.4%; 99% CI, 41.1%-41.8% vs 52.5%; 99% CI, 52.4%-52.5%), compared with nonusers.

**Conclusions and Relevance:**

In this cross-sectional study, we found that acupuncture utilization among patients with LBP was rare but increased over time. Demographic, socioeconomic, and clinical characteristics were associated with acupuncture utilization, and acupuncture users were more likely to utilize other nonpharmacologic treatments and less likely to utilize pharmacologic treatments.

## Key Points

**Question:**

What are the demographic characteristics and pain care regimens of patients with low back pain (LBP) who are able to use their insurance to pay for acupuncture?

**Findings:**

In this cross-sectional study, the share of patients with LBP who had 1 or more insurance claim for acupuncture increased from 0.9% in 2010 to 1.6% in 2019. Acupuncture users were more likely to be high income and college educated, and were less likely to utilize pharmacologic treatments, including opioids.

**Meaning:**

These results show that more patients with LBP are using their insurance to pay for acupuncture but it remains rare, which could be related to financial and nonfinancial constraints, LBP severity, referral management, and patient preferences.

## Teaser

This cross-sectional study examines trends in use of acupuncture to treat low back pain, both in overall utilization of acupuncture and electroacupuncture and associations with demographic and clinical characteristics.

## Introduction

Low back pain (LBP) is a leading cause of disability.^1^ Treating it safely and effectively remains a challenge, often involving a combination of pharmacologic, nonpharmacologic, and interventional therapies.^2,3^ Barriers in access to LBP care are well documented, and are driven by a shortage of pain care clinicians, lack of training in pain care, the time and out-of-pocket costs associated with nonpharmacologic and interventional treatments, and low rates of insurance coverage for certain modalities.^4,5,6,7^

Ensuring that patients with LBP have adequate access to safer pain care is pressing given the ongoing reduction in opioid prescribing, which is disproportionately affecting patients with moderate to severe pain and is associated with increased adverse events, including illicit opioid use, mental health crises, and suicide.^8,9,10^ A postopioid model of pain care, which emphasizes nonpharmacologic approaches like physical therapy and nonsteroidal anti-inflammatory drugs (NSAIDs), is critical for patients who previously relied on opioids.^11,12,13,14^

Acupuncture therapy can play an important role in pain care, and multiple systematic reviews and meta-analyses have demonstrated its effectiveness for treating LBP.^15,16,17,18,19,20^ The American College of Physicians and the American Academy of Family Physicians recommend acupuncture as one first-line option for acute, subacute, and chronic LBP,^2,21,22^ while the US Agency for Healthcare Research and Quality (AHRQ), US Department of Health & Human Services (HHS), and Joint Commission recommend acupuncture as part of comprehensive pain care options.^2,23,24,25^ Yet little is known about the utilization of acupuncture therapy within pain care regimens for LBP, and few studies have leveraged insurance claims to track trends and characteristics associated with acupuncture utilization at the population level.

In this study, we use a large insurance claims database in the US to measure the utilization of reimbursed acupuncture therapy among a sample of insured adults with LBP between 2010 and 2019. In addition to measuring acupuncture utilization and demographic, socioeconomic, and clinical characteristics associated with use, we track how adults with LBP utilize acupuncture therapy in conjunction with pharmacologic treatments (eg, opioids, gabapentinoids), nonpharmacologic treatments (eg, physical therapy, chiropractic care), and interventional treatments (eg, epidural steroid injections).

## Methods

This claims-based study was deemed exempt by the institutional review board at the University of Pennsylvania and followed the Strengthening the Reporting of Observational Studies in Epidemiology (STROBE) guidelines for cross-sectional studies.

Our primary data source was Optum’s de-identified Clinformatics DataMart Database, which includes patients with Medicare Advantage and commercial insurance, including employer-sponsored coverage, and contains insurance claims for every reimbursed office visit, procedure, and prescription fill. We identified patients with 1 or more claim indicating LBP in a given year, which amounted to a repeated cross-sectional analysis. Prior to 2015, LBP was captured by the *International Classification of Diseases, Ninth Edition* (*ICD-9*) code 724.2. After 2015, we used the *International Statistical Classification of Diseases and Related Health Problems, Tenth Revision *(*ICD-10*) code M54.5. Our study sample excluded patients with any missing demographic information (age, sex, race and ethnicity, or region) and patients who were aged under 18 years.

We identified acupuncture claims using Current Procedural Terminology (CPT) codes 97810 and 97811. We also identified electroacupuncture claims, which involve the electrical stimulation of inserted acupuncture needles, using CPT codes 97813 and 97814. Other categories of nonpharmacologic pain care, including physical therapy, chiropractic care, psychotherapy, and occupational therapy, were identified using CPT codes. We captured 5 categories of pharmacologic treatments (antidepressants, gabapentinoids, muscle relaxants, NSAIDs, and opioids) using generic drug names. Finally, we measured 4 categories of interventional treatments, again using CPT codes: facet joint interventions, epidural steroid injections, trigger point injections, and spinal cord stimulators. Nonpharmacologic treatments tend to be more conservative, while interventional treatments rely on diagnostic blockades, regional anesthesia, ablation procedures, and, in rare circumstances, surgical interventions.^26,27^ For all categories, we generated a binary indicator of whether a patient had a claim indicating 1 or more procedure or prescription fill in a given year. Groups of CPT codes, which were identified using the Medicare Fee Schedule and Medicare Coverage Database, and generic drug names, which were identified using the American Hospital Formulary Service Pharmacologic-Therapeutic Classification System, are available in the appendix (see eTables 1 through 3 in Supplement 1).^28,29,30,31,32^

Demographic characteristics included sex (male, female), race and ethnicity (non-Hispanic Asian, non-Hispanic Black, Hispanic, non-Hispanic White, following categorization in the claims database), and age. Socioeconomic characteristics included annual household income (under $40 000, $40 000 to $59 999, $60 000 to $74 999, $75 000 to $99 999, $100 000 or more) and educational attainment (high school graduate or less, some college including an associate’s degree, college graduate). We were able to account for residence in 9 census regions: New England (Connecticut, Maine, Massachusetts, New Hampshire, Rhode Island, Vermont), Middle Atlantic (New Jersey, New York, Pennsylvania), East North Central (Illinois, Indiana, Michigan, Ohio, Wisconsin), West North Central (Iowa, Kansas, Minnesota, Missouri, Nebraska, North Dakota, South Dakota), South Atlantic (Delaware, Washington, DC, Florida, Georgia, Maryland, North Carolina, South Carolina, Virginia, West Virginia), East South Central (Alabama, Kentucky, Mississippi, Tennessee), West South Central (Arkansas, Louisiana, Oklahoma, Texas), Mountain (Arizona, Colorado, Idaho, Montana, Nevada, New Mexico, Utah, Wyoming), and Pacific (Alaska, California, Hawaii, Oregon, Washington). The claims database did not include individuals residing in US territories.

We generated the following clinical characteristics: an indicator for whether the patient had chronic LBP, defined as having 2 or more claims indicating LBP that occurred over 90 days apart, an indicator for whether the patient had a pregnancy that coincided with their LBP diagnosis, an indicator for whether a patient had cancer that coincided with their LBP diagnosis, and a Charlson Comorbidity Index measure.^33^

### Statistical Analysis

First, we plotted trends in the share of patients that had 1 or more insurance claim for acupuncture or electroacupuncture, along with 99% confidence internals. Among acupuncture and electroacupuncture users, we measured the number of visits within a calendar year. We defined this as the number of unique days with 1 or more CPT code of 97810 or 97813, which indicates the first 15 minutes of a given acupuncture or electroacupuncture visit. In supplemental analyses, we estimated trends and 99% confidence intervals by sex, race and ethnicity, education, annual household income, and for patients who reside in the Pacific, Middle Atlantic, and South Atlantic, which were the 3 most populated census regions among acupuncture users.

Next, we compared characteristics for patients with 1 or more claim for acupuncture or electroacupuncture and patients without any claims for acupuncture or electroacupuncture, who we refer to as nonusers. To identify which characteristics were associated with acupuncture and electroacupuncture use, we estimated logistic regressions at the patient-year level. The outcome variable was a binary indicator of whether patients had 1 or more claim for acupuncture or electroacupuncture in a given year. We controlled for demographic characteristics, socioeconomic characteristics, clinical characteristics, region, and year. Standard errors were clustered at the patient level.

Finally, we compared differences in the use of other nonpharmacologic, pharmacologic, and interventional treatments between acupuncture and/or electroacupuncture users and nonusers. Treatments could occur before or after the acupuncture and/or electroacupuncture claim occurred. Differences were deemed statistically significant if the confidence intervals did not overlap—given the large sample size, we again used 99% confidence intervals. Because the sample of users was smaller and had meaningful differences than nonusers, we included least-square (ie, marginal) means, which adjust for age, sex, race and ethnicity, annual household income, educational attainment, clinical characteristics, region, and year. In supplemental analyses, we repeated all analyses for patients with chronic LBP, patients with Medicare Advantage, and patients with commercial insurance.

## Results

Our study sample included 6 840 497 adults with 1 or more diagnosis of LBP between 2010 and 2019, which amounted to 11 227 253 patient-year observations (mean [SD] age, 54.6 [17.8] years; 3 916 766 female [57.3%]; 802 579 Hispanic [11.7%], 258 087 non-Hispanic Asian [3.8%], 804 975 non-Hispanic Black [11.8%], 4 974 856 non-Hispanic White [72.7%]). Approximately a quarter of patients (1 759 243 [25.7%]) had an annual household income of $40 000 or less, a quarter (1 823 104 [26.7%]) had an income over $100 000, and over 70% (4 887 734 [71.5%]) had some college education. Patients were most likely to reside in the South Atlantic (1 799 243 [26.3%]), followed by the West South Central (985 784 [14.4%]) and East North Central (934 583 [13.7%]) regions. Roughly one-third (2 368 422 [34.6%]) had chronic LBP, while 14.5% (992 081 patients) had LBP coincide with cancer and 2.4% (165 881 patients) had LBP coincide with a pregnancy. We excluded 243 319 patients who were under age 18 years and another 469 208 patients who were missing demographic information.

**Figure 1.**
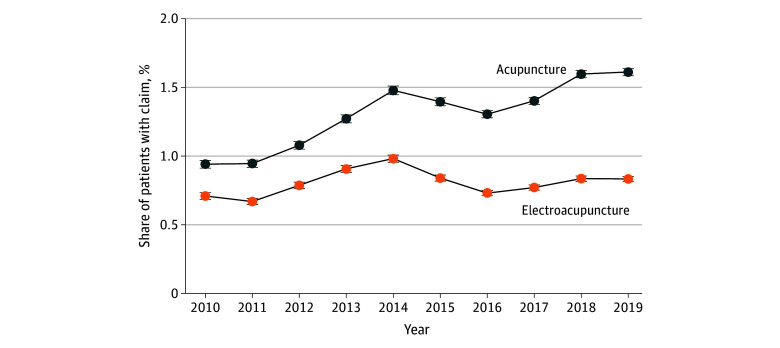
Share of Patients With Low Back Pain With Any Acupuncture or Electroacupuncture Claim, 2010-2019 Error bars indicate 99% CIs.

Overall, 106 485 patients with LBP (1.6%) had 1 or more acupuncture claim, while 61 503 (0.9%) had 1 or more electroacupuncture claim; 29 217 patients (0.4%) had both an acupuncture and electroacupuncture claim. The annual rate of acupuncture use increased consistently, from 0.9% (7505 patients) in 2010 to 1.6% (24 450 patients) in 2019 (Figure 1). Trends in electroacupuncture use were relatively stable: 0.7% (5666 patients) in 2010 and 0.8% (12 645 patients) in 2019. Findings were similar in most subgroups (eFigures 1 through 4 in Supplement 1).

**Figure 2.**
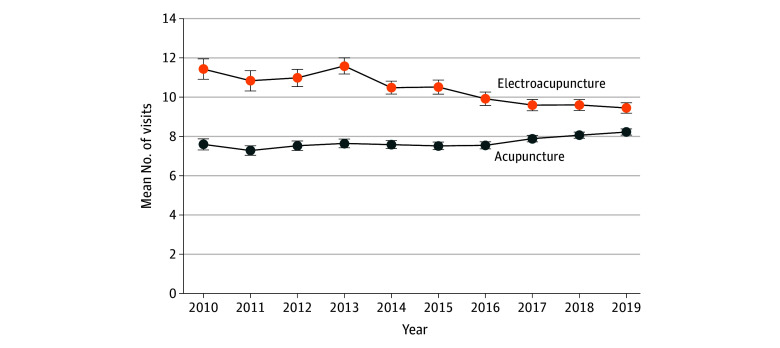
Mean Number of Visits Among Patients With Low Back Pain With Any Acupuncture or Electroacupuncture Claim, 2010-2019 Error bars indicate 99% CIs.

Among those who utilized acupuncture, there was a modest but statistically significant increase in the mean (SD) number of visits per year, from 7.6 (9.4) in 2010 to 8.2 (9.3) in 2019 (Figure 2). For electroacupuncture, the mean (SD) number of visits fell from 11.4 (15.1) in 2010 to 9.5 (11.4) in 2019.

Patients with LBP who utilized acupuncture and electroacupuncture were more likely to be Asian and less likely to be Black, Hispanic, or White compared with nonusers (Table 1). Acupuncture and electroacupuncture users were younger on average (mean [SD] age: acupuncture, 46.9 [15.2] years; electroacupuncture, 48.0 [15.2] years; nonusers, 54.7 [17.9] years), more likely to be female (acupuncture, 69 127 of 106 485 patients [64.9]; electroacupuncture, 37 469 of 61 503 patients [60.9%]; nonusers, 3 828 646 of 6 701 726 patients [57.1%]), most likely to reside in the Pacific region (acupuncture, 45 648 of 106 485 patients [42.9%]; electroacupuncture, 32 399 of 61 503 patients [52.7%]; nonusers, 705 957 of 6 701 726 [10.5%]), had higher incomes (over $100 000: acupuncture, 52 113 of 106 485 patients [48.9%]; electroacupuncture, 31 424 of 61 503 patients [51.1%]; nonusers, 1 754 737 of 6 701 726 [26.2%]), and had more educational attainment than nonusers.

**Table 1.** Characteristics of Patients With Low Back Pain (LBP), 2010-2019

Characteristic	Patients, No. (%)		
	Used acupuncture (n = 106 485)	Used electroacupuncture (n = 61 503)	Never used acupuncture or electroacupuncture (n = 6 701 726)
Age, mean (SD), y	46.9 (15.2)	48.0 (15.2)	54.7 (17.9)
Race and ethnicity			
Hispanic	12 185 (11.4)	6792 (11.0)	786 718 (11.7)
Non-Hispanic Asian	21 843 (20.5)	22 162 (36.0)	224 938 (3.4)
Non-Hispanic Black	4798 (4.5)	2379 (3.9)	798 835 (11.9)
Non-Hispanic White	67 659 (63.5)	30 170 (49.1)	4 891 235 (73.0)
Sex			
Female	69 127 (64.9)	37 469 (60.9)	3 828 646 (57.1)
Male	37 358 (35.1)	24 034 (39.1)	2 873 080 (42.9)
Clinical characteristics			
Chronic LBP	64 076 (60.2)	38.018 (61.8)	2 287 104 (34.1)
Any cancer	14 154 (13.3)	7923 (12.9)	973 936 (14.5)
Any pregnancy	9337 (8.8)	4407 (7.2)	154 814 (2.3)
Insurance type			
Commercial	91 556 (86.0)	51 615 (83.9)	4 099 044 (61.6)
Medicare Advantage	14 997 (14.0)	9921 (16.1)	2 639 570 (39.4)
Annual household income, $			
<40 000	12 847 (12.1)	6875 (11.2)	1 742 807 (26.0)
40 000-59 999	10 109 (9.5)	5567 (9.0)	1 028 983 (15.4)
60 000-74 999	8804 (8.3)	4985 (8.1)	667 247 (10.0)
75 000-99 999	13 852 (13.0)	7419 (12.1)	931 523 (13.9)
≥100 000	52 113 (48.9)	31 424 (51.1)	1 754 737 (26.2)
Missing	8760 (8.2)	5233 (8.5)	576 429 (8.6)
Educational attainment			
High school or less	10 521 (9.9)	6594 (10.7)	1 892 519 (28.2)
Some college	48 796 (45.8)	25 712 (41.8)	3 655 794 (54.6)
College	46 615 (43.8)	28 956 (47.1)	1 108 087 (16.5)
Missing	553 (0.5)	241 (0.4)	45 326 (0.7)
Region			
East North Central	4303 (4.0)	1805 (2.9)	929 161 (13.9)
East South Central	489 (0.5)	216 (0.4)	350 714 (5.2)
Middle Atlantic	16 986 (16.0)	11 853 (19.3)	452 461 (6.8)
Mountain	8788 (8.3)	3338 (5.4)	660 227 (9.9)
New England	2880 (2.7)	737 (1.2)	225 934 (3.4)
Pacific	45 648 (42.9)	32 399 (52.7)	705 957 (10.5)
South Atlantic	12 562 (11.8)	5977 (9.7)	1 783 426 (26.6)
West North Central	8073 (7.6)	2015 (3.3)	673 539 (10.1)
West South Central	8080 (7.6)	3851 (6.3)	975 210 (14.6)

Logistic regressions showed that Black patients (odds ratio [OR], 0.88; 99% CI, 0.84-0.92) were less likely to utilize acupuncture than White patients, while Hispanic patients (OR, 1.23; 99% CI, 1.19-1.27) were more likely to utilize acupuncture than White patients. All 3 groups were substantially less likely to utilize acupuncture than Asian patients (OR, 3.26; 99% CI, 3.18-3.35) (Table 2). Patients who were aged 35 to 44 years were more likely than other age groups to utilize acupuncture (OR, 5.22; 99% CI, 4.99-5.45). Patients who had chronic LBP (OR, 2.39; 99% CI, 2.35-2.43), had cancer (OR, 1.31; 99% CI, 1.27-1.35), or had a pregnancy (OR, 1.89; 99% CI, 1.82-1.96) were more likely to engage in acupuncture, but their clinical complexity, as captured by the Charlson Comorbidity Index, was negatively correlated with acupuncture utilization (OR, 0.92; 99% CI, 0.91-0.92). There were associations of acupuncture use with income (under $40 000 vs $100 000 or higher: OR, 0.59; 99% CI, 0.57-0.61), education (high school or less vs college: OR, 0.32; 95% CI, 0.27-0.35), and the likelihood of using insurance to pay for acupuncture. Patients who were college graduates and from households with an annual income of over $100 000 were most likely to utilize acupuncture compared with other education and income groups. The factors associated with electroacupuncture were similar, except there was no significant difference in use between non-Hispanic Black patients and non-Hispanic White patients.

**Table 2.** Logistic Regressions of Characteristics Associated With Any Acupuncture or Electroacupuncture Use Among Patients With Low Back Pain, 2010-2019^a^

Variable	Any acupuncture^b^		Any electroacupuncture^b^	
	OR (99% CI)	*P* value	OR (99% CI)	*P* value
Age, y				
≥75	1 [Reference]	[Reference]	1 [Reference]	[Reference]
18-24	2.85 (2.68-3.03)	<.001	2.00 (1.83-2.17)	<.001
25-34	5.03 (4.81-5.27)	<.001	3.12 (2.94-3.32)	<.001
35-44	5.22 (4.99-5.45)	<.001	3.58 (3.39-3.79)	<.001
45-54	4.17 (4.00-4.36)	<.001	3.33 (3.15-3.52)	<.001
55-64	3.09 (2.96-3.23)	<.001	2.56 (2.42-2.71)	<.001
65-74	1.61 (1.54-1.68)	<.001	1.50 (1.42-1.59)	<.001
Race and ethnicity				
Non-Hispanic White	1 [Reference]	[Reference]	1 [Reference]	[Reference]
Hispanic	1.23 (1.19-1.27)	<.001	1.45 (1.39-1.51)	<.001
Non-Hispanic Asian	3.26 (3.18-3.35)	<.001	8.06 (7.82-8.30)	<.001
Non-Hispanic Black	0.88 (0.84-0.92)	<.001	1.01 (0.94-1.08)	.80
Sex				
Female	1 [Reference]	[Reference]	1 [Reference]	[Reference]
Male	0.68 (0.67-0.70)	<.001	0.83 (0.81-0.85)	<.001
Clinical characteristics				
Chronic low back pain	2.39 (2.35-2.43)	<.001	2.62 (2.57-2.68)	<.001
Any cancer	1.31 (1.27-1.35)	<.001	1.23 (1.18-1.28)	<.001
Any pregnancy	1.89 (1.82-1.96)	<.001	1.38 (1.31-1.46)	<.001
Charlson Index	0.92 (0.91-0.92)	<.001	0.91 (0.90-0.92)	<.001
Annual household income, $				
≥100 000	1 [Reference]	[Reference]	1 [Reference]	[Reference]
<40 000	0.59 (0.57-0.61)	<.001	0.56 (0.53-0.58)	<.001
40 000-59 999	0.74 (0.71-0.76)	<.001	0.68 (0.65-0.72)	<.001
60 000-74 999	0.84 (0.81-0.87)	<.001	0.78 (0.74-0.82)	<.001
75 000-99 999	0.87 (0.84-0.89)	<.001	0.81 (0.77-0.84)	<.001
Missing	0.74 (0.71-0.77)	<.001	0.69 (0.66-0.72)	<.001
Educational attainment				
College	1 [Reference]	[Reference]	1 [Reference]	[Reference]
High school or less	0.32 (0.27-0.35)	<.001	0.42 (0.40-0.44)	<.001
Some college	0.52 (0.51-0.53)	<.001	0.53 (0.52-0.55)	<.001
Missing	0.65 (0.57-0.74)	<.001	0.61 (0.50-0.74)	<.001
Region				
Pacific	1 [Reference]	[Reference]	1 [Reference]	[Reference]
East North Central	0.09 (0.08-0.09)	<.001	0.06 (0.06-0.07)	<.001
East South Central	0.03 (0.03-0.04)	<.001	0.03 (0.02-0.03)	<.001
Middle Atlantic	0.66 (0.64-0.68)	<.001	0.70 (0.68-0.73)	<.001
Mountain	0.24 (0.23-0.25)	<.001	0.15 (0.14-0.16)	<.001
New England	0.26 (0.25-0.28)	<.001	0.11 (0.10-0.12)	<.001
South Atlantic	0.14 (0.14-0.15)	<.001	0.11 (0.11-0.12)	<.001
West North Central	0.20 (0.19-0.20)	<.001	0.08 (0.08-0.09)	<.001
West South Central	0.16 (0.16-0.17)	<.001	0.13 (0.12-0.13)	<.001

Abbreviation: OR, odds ratio.

^a^ Year fixed effects are included but not reported. Standard errors are clustered at the patient level.

^b^ 11 227 253 patient-years included in sample.

Acupuncture and/or electroacupuncture users differed in their use of pain care (Table 3). Many differences attenuated when controlling for demographic, socioeconomic, and clinical characteristics. Compared with nonusers, those utilizing acupuncture were more likely to engage in physical therapy (39.2%; 99% CI, 38.9%-39.5% vs 29.3%; 99% CI, 29.3%-29.3%) and were twice as likely to receive chiropractic care (45.1%; 99% CI, 44.7%-45.3% vs 23.1%; 99% CI, 23.1%-23.1%) and psychotherapy (18.5%; 99% CI, 18.3%-18.7% vs 8.3%; 99% CI, 8.3%-8.4%). Conversely, acupuncture and/or electroacupuncture users were less likely to utilize pharmacologic treatments. Of note, opioids were the most common type of LBP care used overall—across the study period, 41.4% (99% CI, 41.1%-41.8%) of acupuncture and/or electroacupuncture users had 1 or more opioid prescription fill, compared with 52.5% (99% CI, 52.4%-52.5%) of nonusers. The use of most interventional treatments was similar across groups, although acupuncture and/or electroacupuncture users were more likely to receive trigger point injections.

**Table 3.** Use of Nonpharmacologic, Pharmacologic, and Interventional Treatments Among Patients With Low Back Pain Disaggregated by Acupuncture and/or Electroacupuncture Use, 2010-2019

Treatment	Unadjusted mean share, % (99% CI) (N = 6 840 497)		*P* value	Least-square mean share, % (99% CI) (N = 6 840 497)^a^		*P* value
	Users^b^	Nonusers		Users^b^	Nonusers	
Nonpharmacologic						
Chiropractic care	45.1 (44.7-45.3)	23.1 (23.1-23.1)	<.001	40.9 (40.7-41.2)	21.0 (20.9-21.0)	<.001
Occupational therapy	3.4 (3.3-3.6)	3.6 (3.6-3.6)	.004	3.8 (3.7-3.9)	3.2 (3.2-3.2)	<.001
Physical therapy	39.2 (38.9-39.5)	29.3 (29.3-29.3)	<.001	40.2 (40.0-40.5)	27.8 (27.7-27.9)	<.001
Psychotherapy	18.5 (18.3-18.7)	8.3 (8.3-8.4)	.004	17.1 (16.9-17.2)	7.4 (7.4-7.5)	<.001
Pharmacologic						
Antidepressant	25.5 (25.2-25.8)	30.8 (30.7-30.8)	<.001	20.9 (20.6-21.3)	24.7 (24.7-24.8)	<.001
Gabapentinoid	13.8 (13.6-14.1)	18.1 (18.1-18.1)	<.001	13.2 (12.9-13.4)	16.2 (16.2-16.3)	<.001
Muscle relaxant	27.0 (26.7-27.2)	33.8 (33.8-33.9)	<.001	20.8 (20.4-21.0)	32.8 (32.7-32.9)	<.001
NSAID	37.7 (37.4-38.0)	41.5 (41.5-41.6)	<.001	38.5 (38.1-38.8)	43.5 (43.4-43.6)	<.001
Opioid	41.4 (41.1-41.8)	52.5 (52.4-52.5)	<.001	38.8 (38.5-39.2)	48.5 (48.4-48.6)	<.001
Interventional						
Epidural steroid injection	11.3 (11.0-11.5)	11.4 (11.4-11.4)	.08	11.2 (10.9-11.4)	9.5 (9.5-9.6)	<.001
Facet joint interventions	5.1 (4.9-5.2)	4.7 (4.7-4.7)	<.001	4.8 (4.7-5.0)	3.9 (3.8-3.9)	<.001
Trigger point injection	7.3 (7.1-7.4)	4.1 (4.1-4.2)	<.001	6.9 (6.8-7.1)	3.6 (3.6-3.7)	<.001
Spinal cord stimulator	0.4 (0.3-0.4)	0.4 (0.4-0.4)	.07	0.3 (0.3-0.4)	0.3 (0.3-0.3)	.79

Abbreviation: NSAID, nonsteroidal anti-inflammatory drug.

^a^ Least-square means are adjusted for demographic characteristics, socioeconomic characteristics, clinical characteristics, region, and year.

^b^ Users include patients with LBP who had 1 or more claim for acupuncture or electroacupuncture.

Results were largely similar for patients with chronic LBP (eTables 4-6 and eFigures 5-6 in Supplement 1), patients with Medicare Advantage (eTables 7-9 and eFigures 7-8 in Supplement 1), and patients with commercial insurance (eTables 10-12 and eFigures 9-10 in Supplement 1). Notably, patients with Medicare Advantage utilized acupuncture at lower rates.

## Discussion

Acupuncture therapy is recommended by the American College of Physicians and the American Academy of Family Physicians as a first-line option for acute, subacute, and chronic LBP, while the AHRQ, HHS, and Joint Commission recommend acupuncture as part of comprehensive pain care.^2,21,22,23,24,25^ How acupuncture therapy is employed within a broader pain care regimen is unclear, in part because few studies have leveraged insurance claims. Exceptions include Larson et al,^34^ who used outpatient claims from TRICARE and found that acupuncture was rarely used in episodes of new LBP; Whedon et al,^35^ who used an all-payer claims database from New Hampshire and found that acupuncturists were 77% less likely to be reimbursed than primary care providers; and Dhruva et al,^36^ who tracked acupuncture using insurance claims but focused on the use of spinal cord stimulators.

No study, to our knowledge, has previously leveraged a national sample of insurance claims to study acupuncture utilization and how it is combined with other treatments for LBP care. We found an increase between 2010 and 2019 in the number of patients with LBP who utilize acupuncture, which aligns with prior research.^37,38^ We also found that demographic, socioeconomic, and clinical characteristics were associated with acupuncture utilization. Specifically, Asian patients, female patients, middle-aged patients, patients whose household had an income over $100 000 annually, patients who were college educated, and patients with chronic LBP were more likely to utilize acupuncture. Electroacupuncture rates were relatively stable, and factors associated with electroacupuncture were similar as acupuncture.

Interesting patterns emerged from our examination of other nonpharmacologic, pharmacologic, and interventional treatments utilized by acupuncture users compared with nonusers. Acupuncture users were much more likely to engage in physical therapy, chiropractic care, and psychotherapy, were less likely to use pharmacologic treatments like opioids and gabapentinoids, and were similarly likely to use most interventional treatments.

While acupuncture utilization increased over time, over 98% of patients with LBP never used acupuncture therapy. This is notable given the increased need for pain care that avoids the liabilities of opioids, which remained the most common treatment among patients with LBP in our sample. Acupuncture has been cited as a potential option for patients who previously relied on opioids, and another claims-based study found initial visits to chiropractors, physical therapists, or acupuncturists for newly onset LBP decreased opioid prescribing.^13,39^

While insurance coverage for acupuncture is increasing, low uptake of acupuncture therapy may be driven by less prevalent or generous insurance coverage.^4,40^ The 2020 decision for Medicare Part B to reimburse acupuncture for LBP suggests that this trend of increasing insurance coverage will continue, as Medicare’s decisions generally have ripple effects.^41,42,43^ However, some Medicare Advantage plans were covering acupuncture therapy before Medicare Part B’s expansion, and there was an increase in the share of patients with Medicare Advantage who utilized acupuncture during the study period (eFigure 7 in Supplement 1). An important element of Medicare’s decision to cover acupuncture is that only approved Medicare clinicians can bill for services, meaning that most licensed acupuncturists are not able to be reimbursed directly.^42^ Other insurance-related barriers, such as higher cost sharing and restrictions on health indications, can still limit access to reimbursed acupuncture.^4,40^

Low uptake of acupuncture might also be related to the number and/or distribution of acupuncturists in the US, which may create difficulties in finding acupuncturists who are geographically proximate.^44^ Geographic disparities could explain our finding that roughly a quarter of all acupuncture visits occurred in the Pacific region—California has more licensed acupuncturists than any other state.^44^ The reasons behind the inconsistent supply of acupuncturists is unclear, although it is notable that California was one of the first states to license acupuncturists and among the few states where Medicaid covers acupuncture, and has done so for decades.^45^ Oregon recently joined California in expanding Medicaid coverage for acupuncture, which was linked to an increase in acupuncture utilization among Oregon’s Medicaid enrollees.^46^

Differences in the use of pain care could relate to financial and nonfinancial constraints, which are likely larger for acupuncture therapy than for pharmacologic treatments. That patients with LBP who utilized acupuncture had higher household incomes and higher levels of education may indicate that these patients are better able to afford out-of-pocket costs.^4^ There are additional hassle costs associated with in-person visits such as an acupuncture visit, including the need for transportation, childcare, and/or paid time off.

There could be varying preferences for pain care as well, which may relate to differences in knowledge about the safety and efficacy of acupuncture, the proximity of pain care clinicians, and less tangible mechanisms like self-advocacy and perceptions of pain. For example, aversion to pharmacologic treatments could result in more interest in nonpharmacologic treatments.^47^ Referral management to pain care clinicians could also drive the utilization of acupuncture vs other types of pain care.^48^ Studies have shown that acupuncture therapy is rarely recommended for acute LBP by primary care clinicians, even when they are familiar with clinical guidelines.^49^

### Limitations

Our study had important limitations, including the cross-sectional study design, which only captured trends in acupuncture utilization and correlations between acupuncture, demographic, socioeconomic, and clinical characteristics, and other types of pain care by insured patients. We constructed the study sample using 1 or more diagnosis of LBP, which was not verified using medical record review or self-reports, and we were not able to capture relevant clinical characteristics, such as pain severity. Although when we repeated analysis for patients with chronic LBP, findings were similar. We do not account for the pathways that patients with LBP are taking (the initial clinician consulted has been shown to affect downstream utilization^50,51,52^), nor do we account for every pain care modality, including self-management and experimental treatments like atrasenten, which has demonstrated promising findings regarding a reduction in pain events for certain patient populations.^53^

The most important limitation is that we are unable to capture all acupuncture visits—according to 1 nationally representative survey, only 40% to 50% of visits with acupuncturists are covered by insurance.^54^ Thus, our reported estimates serve as a lower bound of total utilization of acupuncture therapy.

## Conclusions

In this cross-sectional study using a large insurance claims database, we found that the utilization of acupuncture increased over time. We also found that female patients, Asian patients, and patients with higher incomes and college educations were more likely to use insurance to pay for acupuncture therapy, which could relate to different preferences for pain care or differences in health literacy and behaviors. More utilization of acupuncture, particularly among patients with a higher socioeconomic status, may point to barriers to pain care that are driven by financial and nonfinancial constraints, such as out-of-pocket spending and difficulties in attending appointments. Given the extent of LBP and the continued liability of opioids, it is critical to help patients engage in safe, effective, and affordable pain care such as acupuncture therapy.

## Notes

Supplement 1. **eTable 1.** Current Procedural Terminology (CPT) Codes for Nonpharmacologic Treatments **eTable 2.** Generic Drug Names for Pharmacologic Treatments **eTable 3.** Current Procedural Terminology (CPT) Codes for Interventional Treatments **eFigure 1.** Share of Patients With Low Back Pain With Any Acupuncture or Electroacupuncture Claim by Sex, 2010-2019 **eFigure 2.** Share of Patients With Low Back Pain With Any Acupuncture or Electroacupuncture Claim by Race/Ethnicity, 2010-2019 **eFigure 3.** Share of Patients With Low Back Pain With Any Acupuncture or Electroacupuncture Claim by Socioeconomic Characteristics, 2010-2019 **eFigure 4.** Share of Patients With Low Back Pain With Any Acupuncture or Electroacupuncture Claim by Region, 2010-2019 **eTable 4.** Characteristics of Patients With Chronic Low Back Pain (cLBP), Stratified by Whether They Had 1 or More Acupuncture or Electroacupuncture Claim, 2010-2019 **eFigure 5.** Share of Patients With Chronic Low Back Pain (cLBP) With Any Acupuncture or Electroacupuncture Claim, 2010-2019 **eFigure 6.** Mean Number of Visits Among Patients with Chronic Low Back Pain (cLBP) with Any Acupuncture or Electroacupuncture Claim, 2010-2019 **eTable 5.** Logistic Regressions Predicting Any Acupuncture Use Among Patients With Chronic Low Back Pain (cLBP), 2010-2019 **eTable 6.** Use of Nonpharmacologic, Pharmacologic, and Interventional Treatments Among Patients With Chronic Low Back Pain (cLBP) Disaggregated by Acupuncture/Electroacupuncture Use, 2010-2019 **eTable 7.** Characteristics of Patients With Low Back Pain and Medicare Advantage, Stratified by Whether They Had 1 or More Acupuncture or Electroacupuncture Claim, 2010-2019 **eFigure 7.** Share of Patients With Low Back Pain and Medicare Advantage With Any Acupuncture or Electroacupuncture Claim, 2010-2019 **eFigure 8.** Mean Number of Visits Among Patients With Low Back Pain and Medicare Advantage With Any Acupuncture or Electroacupuncture Claim, 2010-2019 **eTable 8.** Logistic Regressions Predicting Any Acupuncture Use Among Patients With Low Back Pain and Medicare Advantage, 2010-2019 **eTable 9.** Use of Nonpharmacologic, Pharmacologic, and Interventional Treatments Among Patients With Low Back Pain and Medicare Advantage Disaggregated by Acupuncture/Electroacupuncture Use, 2010-2019 **eTable 10.** Characteristics of Patients With Low Back Pain and Commercial Insurance, Stratified by Whether They Had 1 or More Acupuncture or Electroacupuncture Claim, 2010-2019 **eFigure 9.** Share of Patients With Low Back Pain and Commercial Insurance With Any Acupuncture or Electroacupuncture Claim, 2010-2019 **eFigure 10.** Mean Number of Visits Among Patients With Low Back Pain and Commercial Insurance with Any Acupuncture or Electroacupuncture Claim, 2010-2019 **eTable 11.** Logistic Regressions Predicting Any Acupuncture Use Among Patients With Low Back Pain and Commercial Insurance, 2010-2019 **eTable 12.** Use of Nonpharmacologic, Pharmacologic, and Interventional Treatments among Patients with Low Back Pain and Commercial Insurance Disaggregated by Acupuncture/Electroacupuncture Use, 2010-2019

Supplement 2.
Data Sharing Statement
