# Supplementary material for: Utilization of Reimbursed Acupuncture Therapy for Low Back Pain
Source: JAMA Netw Open. 2024 Aug 29;7(8):e2430906. doi: 10.1001/jamanetworkopen.2024.30906 (PMC11362866; doi:10.1001/jamanetworkopen.2024.30906)
Supplement: Supplement 1. — eTable 1. Current Procedural Terminology (CPT) Codes for Nonpharmacologic Treatments eTable 2. Generic Drug Names for Pharmacologic Treatments eTable 3. Current Procedural Terminology (CPT) Codes for Interventional Treatments eFigure 1. Share of Patients With Low Back Pain With Any Acupuncture or Electroacupuncture Claim by Sex, 2010-2019 eFigure 2. Share of Patients With Low Back Pain With Any Acupuncture or Electroacupuncture Claim by Race/Ethnicity, 2010-2019 eFigure 3. Share of Patients With Low Back Pain With Any Acupuncture or Electroacupuncture Claim by Socioeconomic Characteristics, 2010-2019 eFigure 4. Share of Patients With Low Back Pain With Any Acupuncture or Electroacupuncture Claim by Region, 2010-2019 eTable 4. Characteristics of Patients With Chronic Low Back Pain (cLBP), Stratified by Whether They Had 1 or More Acupuncture or Electroacupuncture Claim, 2010-2019 eFigure 5. Share of Patients With Chronic Low Back Pain (cLBP) With Any Acupuncture or Electroacupuncture Claim, 2010-2019 eFigure 6. Mean Number of Visits Among Patients with Chronic Low Back Pain (cLBP) with Any Acupuncture or Electroacupuncture Claim, 2010-2019 eTable 5. Logistic Regressions Predicting Any Acupuncture Use Among Patients With Chronic Low Back Pain (cLBP), 2010-2019 eTable 6. Use of Nonpharmacologic, Pharmacologic, and Interventional Treatments Among Patients With Chronic Low Back Pain (cLBP) Disaggregated by Acupuncture/Electroacupuncture Use, 2010-2019 eTable 7. Characteristics of Patients With Low Back Pain and Medicare Advantage, Stratified by Whether They Had 1 or More Acupuncture or Electroacupuncture Claim, 2010-2019 eFigure 7. Share of Patients With Low Back Pain and Medicare Advantage With Any Acupuncture or Electroacupuncture Claim, 2010-2019 eFigure 8. Mean Number of Visits Among Patients With Low Back Pain and Medicare Advantage With Any Acupuncture or Electroacupuncture Claim, 2010-2019 eTable 8. Logistic Regressions Predicting Any Acupuncture Use Among Patients [file jamanetwopen-e2430906-s001.pdf]

Supplemental Online Content

Candon M, Nielsen A, Dusek JA, et al. Utilization of reimbursed acupuncture therapy for low back pain. *JAMA Netw Open.* 2024;7(8):e2430906.
doi:10.1001/jamanetworkopen.2024.30906

eTable 1. Current Procedural Terminology (CPT) Codes for Nonpharmacologic Treatments

eTable 2. Generic Drug Names for Pharmacologic Treatments

eTable 3. Current Procedural Terminology (CPT) Codes for Interventional Treatments

eFigure 1. Share of Patients With Low Back Pain With Any Acupuncture or Electroacupuncture Claim by Sex, 2010-2019

eFigure 2. Share of Patients With Low Back Pain With Any Acupuncture or Electroacupuncture Claim by Race/Ethnicity, 2010-2019

eFigure 3. Share of Patients With Low Back Pain With Any Acupuncture or Electroacupuncture Claim by Socioeconomic Characteristics, 2010-2019

eFigure 4. Share of Patients With Low Back Pain With Any Acupuncture or Electroacupuncture Claim by Region, 2010-2019

eTable 4. Characteristics of Patients With Chronic Low Back Pain (cLBP), Stratified by Whether They Had 1 or More Acupuncture or Electroacupuncture Claim, 2010-2019

eFigure 5. Share of Patients With Chronic Low Back Pain (cLBP) With Any Acupuncture or Electroacupuncture Claim, 2010-2019

eFigure 6. Mean Number of Visits Among Patients with Chronic Low Back Pain (cLBP) with Any Acupuncture or Electroacupuncture Claim, 2010-2019

eTable 5. Logistic Regressions Predicting Any Acupuncture Use Among Patients With Chronic Low Back Pain (cLBP), 2010-2019

eTable 6. Use of Nonpharmacologic, Pharmacologic, and Interventional Treatments Among Patients With Chronic Low Back Pain (cLBP) Disaggregated by Acupuncture/Electroacupuncture Use, 2010-2019

eTable 7. Characteristics of Patients With Low Back Pain and Medicare Advantage, Stratified by Whether They Had 1 or More Acupuncture or Electroacupuncture Claim, 2010-2019

eFigure 7. Share of Patients With Low Back Pain and Medicare Advantage With Any Acupuncture or Electroacupuncture Claim, 2010-2019

© 2024 Candon M et al. *JAMA Network Open.*

eFigure 8. Mean Number of Visits Among Patients With Low Back Pain and Medicare Advantage With Any Acupuncture or Electroacupuncture Claim, 2010-2019

eTable 8. Logistic Regressions Predicting Any Acupuncture Use Among Patients With Low Back Pain and Medicare Advantage, 2010-2019

eTable 9. Use of Nonpharmacologic, Pharmacologic, and Interventional Treatments Among Patients With Low Back Pain and Medicare Advantage Disaggregated by Acupuncture/Electroacupuncture Use, 2010-2019

eTable 10. Characteristics of Patients With Low Back Pain and Commercial Insurance, Stratified by Whether They Had 1 or More Acupuncture or Electroacupuncture Claim, 2010-2019

eFigure 9. Share of Patients With Low Back Pain and Commercial Insurance With Any Acupuncture or Electroacupuncture Claim, 2010-2019

eFigure 10. Mean Number of Visits Among Patients With Low Back Pain and Commercial Insurance with Any Acupuncture or Electroacupuncture Claim, 2010-2019

eTable 11. Logistic Regressions Predicting Any Acupuncture Use Among Patients With Low Back Pain and Commercial Insurance, 2010-2019

eTable 12. Use of Nonpharmacologic, Pharmacologic, and Interventional Treatments among Patients with Low Back Pain and Commercial Insurance Disaggregated by Acupuncture/Electroacupuncture Use, 2010-2019

This supplemental material has been provided by the authors to give readers additional information about their work.

© 2024 Candon M et al. *JAMA Network Open.*

eTable 1. Current Procedural Terminology (CPT) Codes for Nonpharmacologic Treatments

**Treatment**	**CPT Codes**
Acupuncture	97810, 97811
Electroacupuncture	97813, 97814
Chiropractic care	98940, 98941, 98942, 98943
Occupational therapy	97165, 97166, 97167, 97168, 97003, 97004
Physical therapy	97161, 97162, 97163, 97164, 97001, 97002
Psychotherapy	90785, 90832, 90833, 90834, 90836, 90837, 90838, 90839, 90840, 90845, 90846, 90847, 90849, 90853, 96150, 96151, 96152, 96153, 96154, 90899, 97532, 90876, 90875, 90880 90901, 90912, 90913, 90804, 90805, 90806, 90807, 90808, 90809, 90810, 90811, 90812, 90813, 90814, 90815, 90816, 90817, 90818, 90819, 90821, 90822, 90823, 90824, 90826, 90827, 90828, 90829

© 2024 Candon M et al. *JAMA Network Open.*

eTable 2. Generic Drug Names for Pharmacologic Treatments

Treatment	Generic names
Antidepressants	isocarboxazid, phenelzine, tranylcypromine, rasagiline, selegiline, desvenlafaxine, duloxetine, levomilnacipran, venlafaxine, milnacipran, citalopram, escitalopram, fluoxetine, fluvoxamine, paroxetine, sertraline, trazodone, vortioxetine, amitriptyline, amoxapine, clomipramine, desipramine, doxepin, imipramine, maprotiline, nortriptyline, protriptyline, trimipramine, bupropion, mirtazapine
Gabapentinoids	gabapentin, pregabalin
Muscle relaxants	cyclobenzaprine, methocarbamol, carisoprodol, baclofen, chlorzoxazone, metaxalone, orphenadrine, tizanidine
Nonsteroidal anti-inflammatory drugs (NSAIDs)	diclofenac, diflunisal, etodolac, fenoprofen, flurbiprofen, ibuprofen, indomethacin, ketoprofen, ketorolac, meclofenamate, mefenamic acid, meloxicam, nabumetone, naproxen, oxaprozin, piroxicam, sulindac, tolmetin, aspirin, choline salicylate, magnesium salicylate, salicylate salts, salicylates general statement, salsalate, sodium salicylate, trolamine salicylate, celecoxib
Opioids	codeine, fentanyl, hydrocodone, hydromorphone, levorphanol, meperidine, methadone, morphine, opium, oxycodone, oxymorphone, remifentanil, sufentanil, tapentadol, tramadol, buprenorphine, butorphanol, nalbuphine, pentazocine, acetaminophen/dihydrocodone

© 2024 Candon M et al. *JAMA Network Open.*

eTable 3. Current Procedural Terminology (CPT) Codes for Interventional Treatments

**Treatment**	**CPT Codes**
Epidural steroid injections	62321, 62323, 64479, 64480, 64483, 64484, 62310, 62311
Facet joint interventions	64451, 64470, 64472, 64475, 64476, 64490, 64491, 64492, 64493, 64494, 64494, 64495, 64622, 64623, 64625, 64626, 64627, 64633, 64634, 64635
Trigger point injections	20552, 20553
Spinal cord stimulator	63650, 63655, 63685

© 2024 Candon M et al. *JAMA Network Open.*

eFigure 1. Share of Patients with Low Back Pain with Any Acupuncture or Electroacupuncture Claim by Sex, 2010-2019

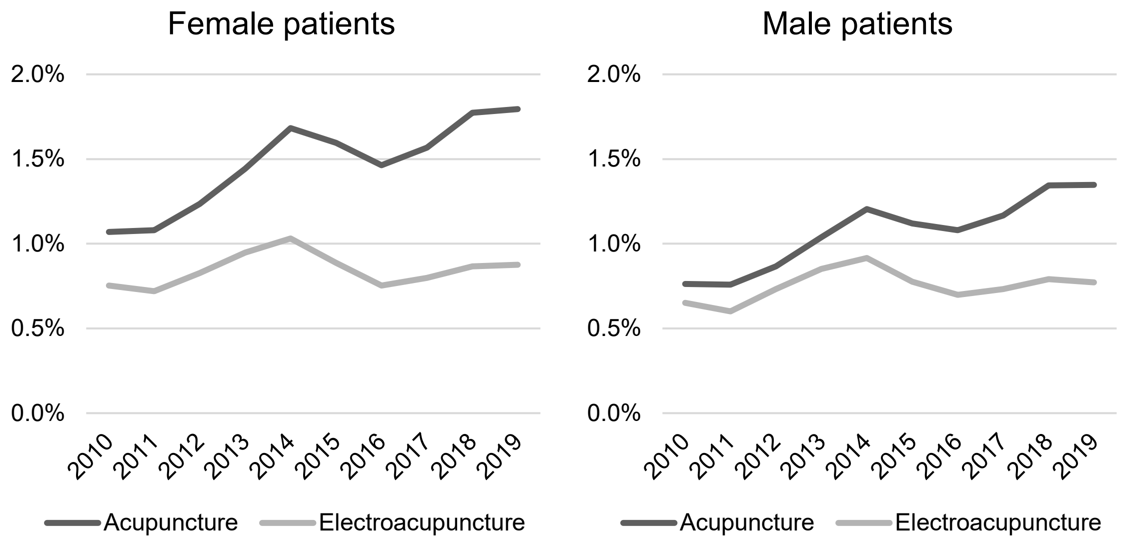

© 2024 Candon M et al. *JAMA Network Open*.

eFigure 2. Share of Patients with Low Back Pain with Any Acupuncture or Electroacupuncture Claim by Race/Ethnicity, 2010-2019

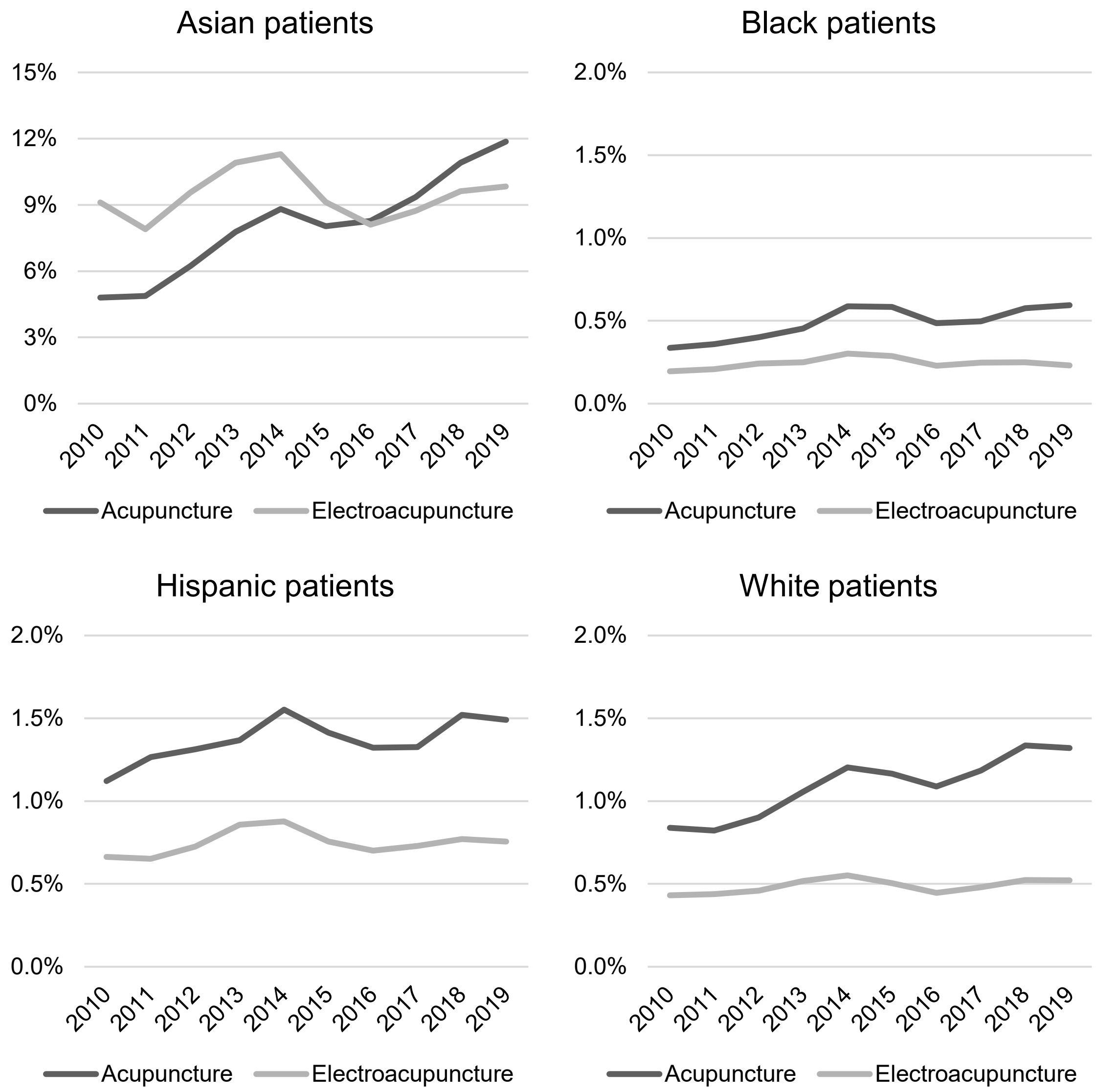

© 2024 Candon M et al. *JAMA Network Open.*

eFigure 3. Share of Patients with Low Back Pain with Any Acupuncture or Electroacupuncture Claim by Socioeconomic Characteristics, 2010-2019

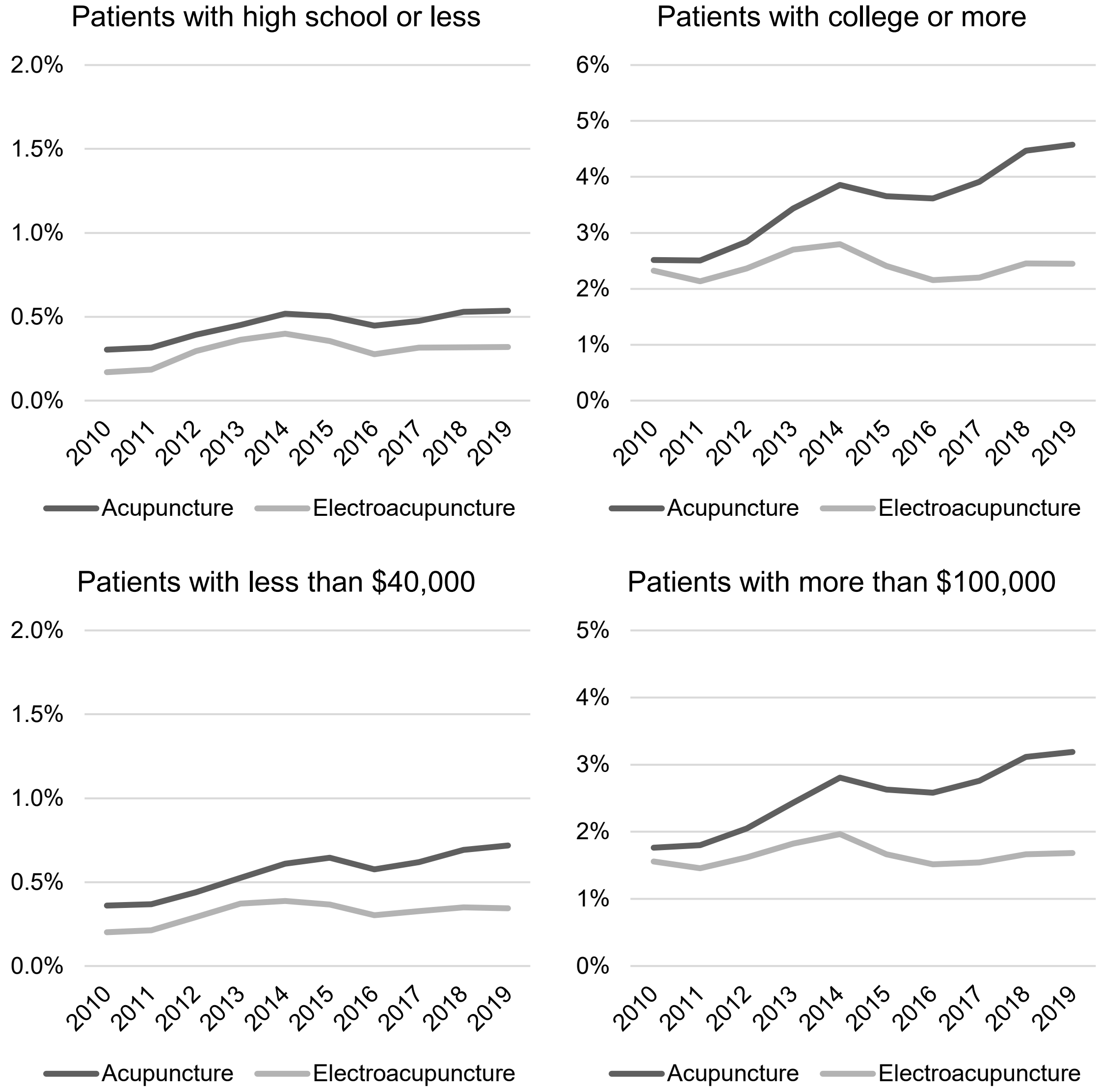

© 2024 Candon M et al. *JAMA Network Open.*

eFigure 4. Share of Patients with Low Back Pain with Any Acupuncture or Electroacupuncture Claim by Region, 2010-2019

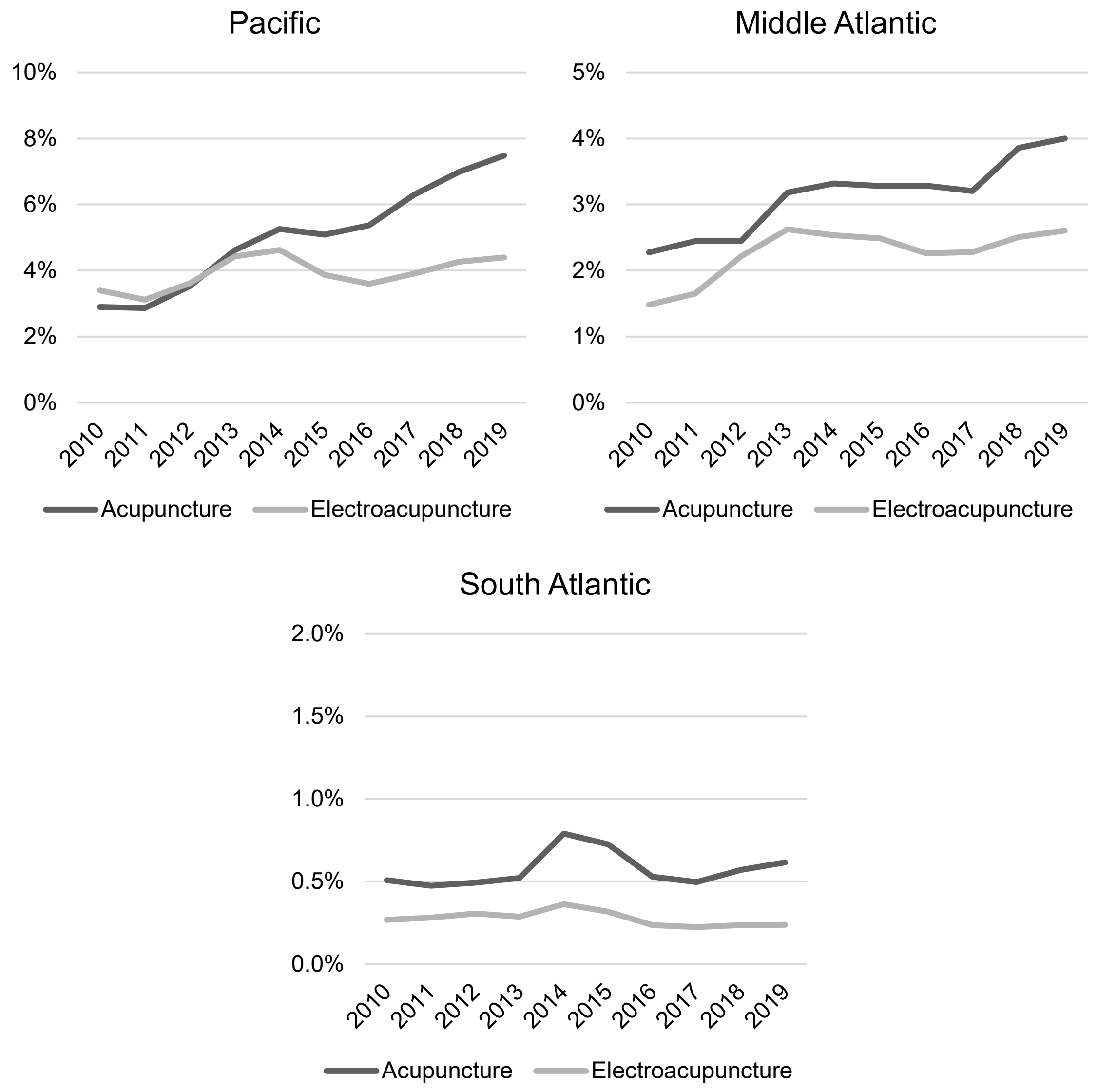

© 2024 Candon M et al. *JAMA Network Open.*

eTable 4. Characteristics of Patients with Chronic Low Back Pain (cLBP), Stratified by Whether They Had 1+ Acupuncture or Electroacupuncture Claim, 2010-2019

	Used acupuncture	Used electroacupuncture	Never used acupuncture or electroacupuncture
	n (%)	n (%)	n (%)
**Age**	48.0 (SD=15.2)	48.7 (SD=15.2)	57.5 (SD=16.8)
**Race/ethnicity**			
Asian	12,277 (21.4)	12,702 (37.0)	61,163 (2.7)
Black	2,543 (4.4)	1,324 (3.9)	271,497 (11.8)
Hispanic	6,406 (11.2)	3,641 (10.6)	238,883 (10.4)
White	36,196 (63.0)	16,711 (48.6)	1,733,460 (75.2)
**Sex**			
Female	37,499 (65.3)	21,122 (61.4)	1,362,290 (59.1)
Male	19,923 (34.7)	13,256 (38.6)	942,713 (40.9)
**Clinical Characteristics**			
Chronic low back pain	57,422 (100.0)	34,378 (100.0)	2,305,003 (100.0)
Any cancer	7,907 (13.8)	4,581 (13.3)	390,572 (16.9)
Any pregnancy	4,610 (8.0)	2,361 (6.9)	41,137 (1.8)
**Annual household income**			
Under $40,000	6,952 (12.1)	3,820 (11.1)	653,190 (28.3)
$40,000-$59,999	5,475 (9.5)	3,077 (9.0)	360,965 (15.7)
$60,000-$74,999	4,783 (8.3)	2,768 (8.1)	230,371 (10.0)
$75,000-$99,999	7,534 (13.1)	4,090 (11.9)	320,308 (13.9)
Over $100,000	28,293 (49.3)	17,850 (51.9)	570,051 (24.7)
Missing	4,385 (7.6)	2,773 (8.1)	170,118 (7.4)
**Educational attainment**			
High school or less	5,657 (9.9)	3,616 (10.5)	672,857 (29.2)
Some college	26,098 (45.5)	14,204 (41.3)	1,259,424 (54.6)
College	25,376 (44.2)	16,427 (47.8)	355,972 (15.4)
Missing	291 (0.5)	131 (0.4)	16,750 (0.7)
**Region**			
East North Central	2,203 (3.8)	963 (2.8)	306,160 (13.3)
East South Central	276 (0.5)	122 (0.4)	128,131 (5.6)
Middle Atlantic	9,448 (16.5)	6,644 (19.3)	145,772 (6.3)
Mountain	4,627 (8.1)	1,824 (5.3)	241,588 (10.5)
New England	1,529 (2.7)	402 (1.2)	72,493 (3.2)

© 2024 Candon M et al. *JAMA Network Open.*

Pacific	24,946 (43.4)	18,582 (54.1)	237,594 (10.3)
South Atlantic	6,738 (11.7)	3,239 (9.4)	635,991 (27.6)
West North Central	4,314 (7.5)	1,123 (3.3)	238,510 (10.4)
West South Central	3,997 (7.0)	1,824 (5.3)	317,602 (13.8)
**Number of Patients**	57,422	34,378	2,305,003

© 2024 Candon M et al. *JAMA Network Open.*

eFigure 5. Share of Patients with Chronic Low Back Pain (cLBP) with Any Acupuncture or Electroacupuncture Claim, 2010-2019

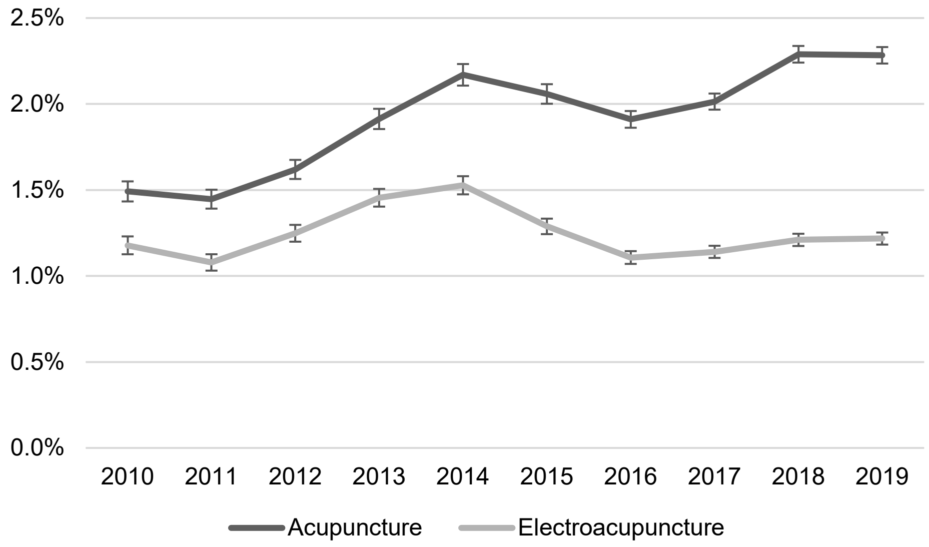

© 2024 Candon M et al. *JAMA Network Open.*

eFigure 6. Mean Number of Visits among Patients with Chronic Low Back Pain (cLBP) with Any Acupuncture or Electroacupuncture Claim, 2010-2019

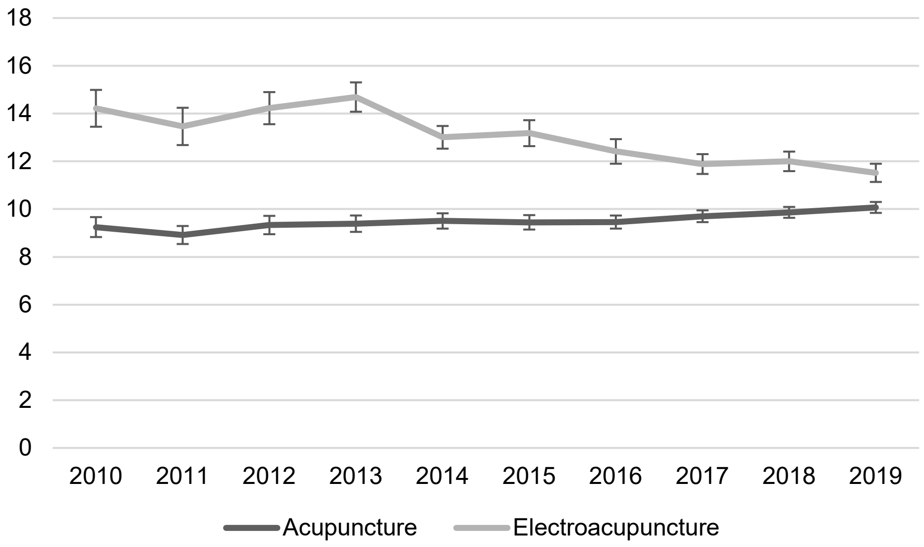

© 2024 Candon M et al. *JAMA Network Open.*

eTable 5. Logistic Regressions Predicting Any Acupuncture Use among Patients with Chronic Low Back Pain (cLBP), 2010-2019

	**Any Acupuncture**		**Any Electroacupuncture**	
	Odds ratio (99% CI)	p-value	Odds ratio (99% CI)	p-value
**Age (reference group: 75+ years)**				
18-24 years	2.83 (2.60-3.10)	<0.001	2.15 (1.90-2.43)	<0.001
25-34 years	5.08 (4.77-5.40)	<0.001	3.31 (3.06-3.59)	<0.001
35-44 years	5.01 (4.73-5.30)	<0.001	3.63 (3.38-3.90)	<0.001
45-54 years	3.92 (3.70-4.15)	<0.001	3.24 (3.02-3.48)	<0.001
55-64 years	2.85 (2.69-3.02)	<0.001	2.43 (2.26-2.61)	<0.001
65-74 years	1.51 (1.43-1.61)	<0.001	1.42 (1.32-1.53)	<0.001
**Race (reference group: White)**				
Asian	3.69 (3.55-3.83)	<0.001	8.87 (8.51-9.25)	<0.001
Black	0.95 (0.89-1.01)	0.031	1.07 (0.98-1.17)	0.037
Hispanic	1.39 (1.33-1.45)	<0.001	1.57 (1.48-1.67)	<0.001
**Sex (reference group: Female)**				
Male	0.69 (0.67-0.71)	<0.001	0.84 (0.82-0.87)	<0.001
**Clinical characteristics**				
Any cancer	1.30 (1.25-1.35)	<0.001	1.22 (1.16-1.29)	<0.001
Any pregnancy	1.84 (1.74-1.94)	<0.001	1.32 (1.22-1.43)	<0.001
Charlson index	0.90 (0.89-0.91)	<0.001	0.90 (0.89-0.91)	<0.001
**Annual household income (reference group: Over $100,000)**				
Under $40,000	0.58 (0.55-0.61)	<0.001	0.55 (0.52-0.58)	<0.001
$40,000-$59,999	0.74 (0.71-0.78)	<0.001	0.67 (0.63-0.72)	<0.001
$60,000-$74,999	0.85 (0.81-0.89)	<0.001	0.78 (0.73-0.83)	<0.001
$75,000-$99,999	0.87 (0.84-0.91)	<0.001	0.80 (0.75-0.85)	<0.001
Missing	0.75 (0.72-0.79)	<0.001	0.70 (0.66-0.75)	<0.001
**Educational attainment (reference group: College)**				
High school or less	0.31 (0.30-0.33)	<0.001	0.41 (0.38-0.43)	<0.001
Some college	0.50 (0.49-0.52)	<0.001	0.52 (0.50-0.54)	<0.001
Missing	0.62 (0.52-0.75)	<0.001	0.56 (0.43-0.73)	<0.001
**Region (reference group: Pacific)**				
East North Central	0.09 (0.08-0.09)	<0.001	0.06 (0.05-0.07)	<0.001
East South Central	0.03 (0.03-0.04)	<0.001	0.03 (0.02-0.03)	<0.001
Middle Atlantic	0.73 (0.70-0.76)	<0.001	0.72 (0.69-0.76)	<0.001
Mountain	0.23 (0.21-0.24)	<0.001	0.14 (0.13-0.15)	<0.001
New England	0.29 (0.26-0.31)	<0.001	0.11 (0.09-0.13)	<0.001

© 2024 Candon M et al. *JAMA Network Open.*

South Atlantic	0.15 (0.14-0.15)	<0.001	0.11 (0.10-0.12)	<0.001
West North Central	0.19 (0.18-0.20)	<0.001	0.08 (0.07-0.09)	<0.001
West South Central	0.16 (0.15-0.17)	<0.001	0.11 (0.11-0.12)	<0.001
**Number of Patient-Years**	4,420,864		4,420,864	

© 2024 Candon M et al. *JAMA Network Open.*

eTable 6. Use of Nonpharmacologic, Pharmacologic, and Interventional Treatments among Patients with Chronic Low Back Pain (cLBP) Disaggregated by Acupuncture/Electroacupuncture Use, 2010-2019

	**Users: Unadjusted mean**	**Non-users: Unadjusted mean**		**Users: Least-square mean**	**Non-users: Least-square mean**	
	share (99% CI)	share (99% CI)	p-value	share (99% CI)	share (99% CI)	p-value
**Nonpharmacologic Treatments**						
Chiropractic care	48.5% (48.1-48.9%)	29.1% (29.0-29.2%)	<0.001	45.1% (44.7-45.5%)	28.9% (28.8-29.0%)	<0.001
Occupational therapy	3.9% (3.7-4.1%)	4.8% (4.7-4.8%)	<0.001	4.1% (3.9-4.3%)	4.0% (3.9-3.1%)	<0.001
Physical therapy	43.1% (42.7-43.6%)	37.7% (37.6-37.8%)	<0.001	44.4% (43.9-44.8%)	35.8% (35.7-36.0%)	<0.001
Psychotherapy	19.8% (19.5-20.1%)	10.3% (10.3-10.4%)	<0.001	18.3% (18.0-18.6%)	10.0% (9.9-10.0%)	<0.001
**Pharmacologic Treatments**						
Antidepressant	26.6% (26.1-27.0%)	38.7% (38.7-38.8%)	<0.001	21.7% (21.2-22.1%)	32.3% (32.2-32.5%)	<0.001
Gabapentinoid	16.0% (15.6-16.4%)	27.7% (27.6-27.7%)	<0.001	14.7% (14.3-15.2%)	24.5% (24.3-24.7%)	<0.001
Muscle relaxant	25.4% (25.0-25.9%)	38.5% (38.4-38.6%)	<0.001	22.1% (21.6-22.5%)	37.9% (37.8-38.1%)	<0.001
NSAID	39.6% (39.1-40.1%)	46.5% (46.4-46.6%)	<0.001	40.7% (40.2-41.1%)	58.8% (48.6-49.0%)	<0.001
Opioid	43.2% (42.7-43.7%)	62.7% (62.6-62.8%)	<0.001	41.0% (40.6-41.5%)	58.2% (58.0-58.3%)	<0.001
**Interventional Treatments**						
Epidural steroid injection	14.4% (14.0-14.8%)	20.4% (20.3-20.5%)	<0.001	14.2% (13.8-14.6%)	16.6% (16.5-16.7%)	0.004
Facet joint interventions	6.9% (6.6-7.2%)	9.7% (9.6-9.7%)	<0.001	6.3% (6.0-6.6%)	7.6% (7.5-7.7%)	<0.001
Trigger point injection	8.9% (8.7-9.2%)	7.2% (7.1-7.2%)	<0.001	8.4% (8.2-8.7%)	6.3% (6.2-6.4%)	<0.001
Spinal cord stimulator	0.5% (0.5-0.6%)	0.9% (0.9-1.0%)	<0.001	0.4% (0.3-0.5%)	0.6% (0.6-0.7%)	0.727
**Number of Patients**	2,378,796			2,378,796		

© 2024 Candon M et al. *JAMA Network Open.*

eTable 7. Characteristics of Patients with Low Back Pain and Medicare Advantage, Stratified by Whether They Had 1+ Acupuncture or Electroacupuncture Claim, 2010-2019

	Used acupuncture	Used electroacupuncture	Never used acupuncture or electroacupuncture
	n (%)	n (%)	n (%)
**Age**	71.6 (SD=7.6)	71.7 (SD=7.1)	70.7 (SD=10.2)
**Race/ethnicity**			
Asian	4,780 (31.9)	4,943 (49.8)	69,716 (2.6)
Black	493 (3.3)	265 (2.7)	362,187 (13.7)
Hispanic	1,281 (8.5)	823 (8.3)	299,185 (11.3)
White	8,443 (56.3)	3,890 (39.2)	1,908,482 (72.3)
**Sex**			
Female	9,532 (63.6)	6,017 (60.7)	1,609,201 (61.0)
Male	5,465 (36.4)	3,904 (39.4)	1,030,369 (39.0)
**Clinical Characteristics**			
Chronic low back pain	10,651 (71.0)	6,497 (65.5)	1,099,646 (41.7)
Any cancer	4,346 (29.0)	2,753 (27.8)	658,327 (24.9)
Any pregnancy	28 (0.2)	14 (0.1)	5,708 (0.2)
**Annual household income**			
Under $40,000	3,127 (20.9)	2,030 (20.5)	999,933 (37.9)
$40,000-$59,999	2,612 (17.4)	1,692 (17.1)	495,379 (18.8)
$60,000-$74,999	1,871 (12.5)	1,285 (13.0)	277,056 (10.5)
$75,000-$99,999	2,542 (17.0)	1,712 (17.3)	332,093 (12.6)
Over $100,000	4,290 (28.6)	2,856 (28.8)	357,928 (13.6)
Missing	555 (3.7)	346 (3.5)	177,181 (6.7)
**Educational attainment**			
High school or less	2,861 (19.1)	2,454 (24.7)	878,383 (33.3)
Some college	8,166 (54.5)	4,882 (49.2)	1,431,875 (54.3)
College	3,909 (26.1)	2,553 (25.7)	308,247 (11.7)
Missing	61 (0.4)	32 (0.3)	21,066 (0.8)
**Region**			
East North Central	672 (4.5)	250 (2.5)	319,264 (12.1)
East South Central	74 (0.5)	43 (0.4)	139,556 (5.3)
Middle Atlantic	3,666 (24.4)	4,221 (42.6)	208,059 (7.9)
Mountain	896 (6.0)	372 (2.8)	265,952 (10.1)
New England	740 (4.9)	158 (1.6)	118,550 (4.5)
Pacific	6,584 (43.9)	4,033 (40.7)	337,671 (12.8)

© 2024 Candon M et al. *JAMA Network Open.*

South Atlantic	1,249 (8.3)	511 (5.2)	734,418 (27.8)
West North Central	838 (5.6)	206 (2.1)	220,757 (8.4)
West South Central	370 (2.5)	169 (1.7)	315,282 (11.9)
**Number of Patients**	14,997	9,921	2,639,570

© 2024 Candon M et al. *JAMA Network Open.*

eFigure 7. Share of Patients with Low Back Pain and Medicare Advantage with Any Acupuncture or Electroacupuncture Claim, 2010-2019

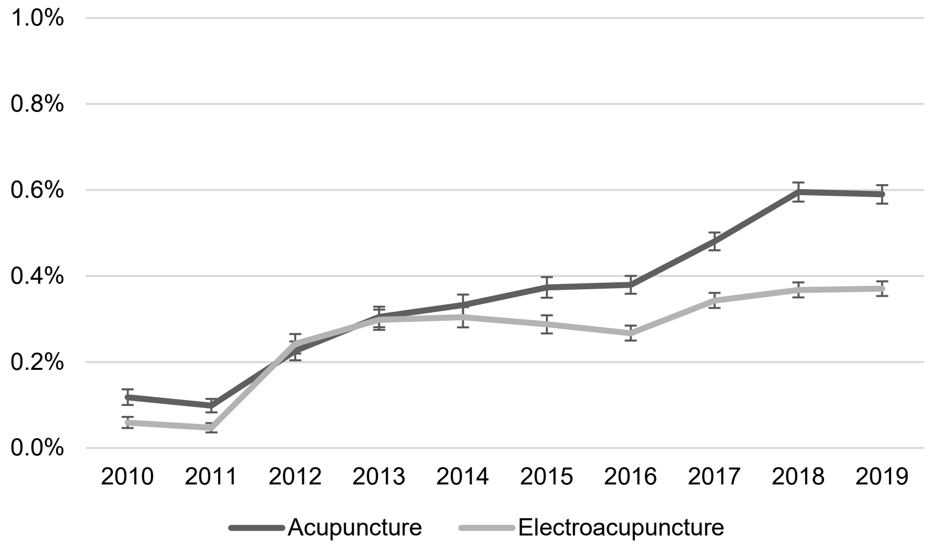

© 2024 Candon M et al. *JAMA Network Open.*

eFigure 8. Mean Number of Visits among Patients with Low Back Pain and Medicare Advantage with Any Acupuncture or Electroacupuncture Claim, 2010-2019

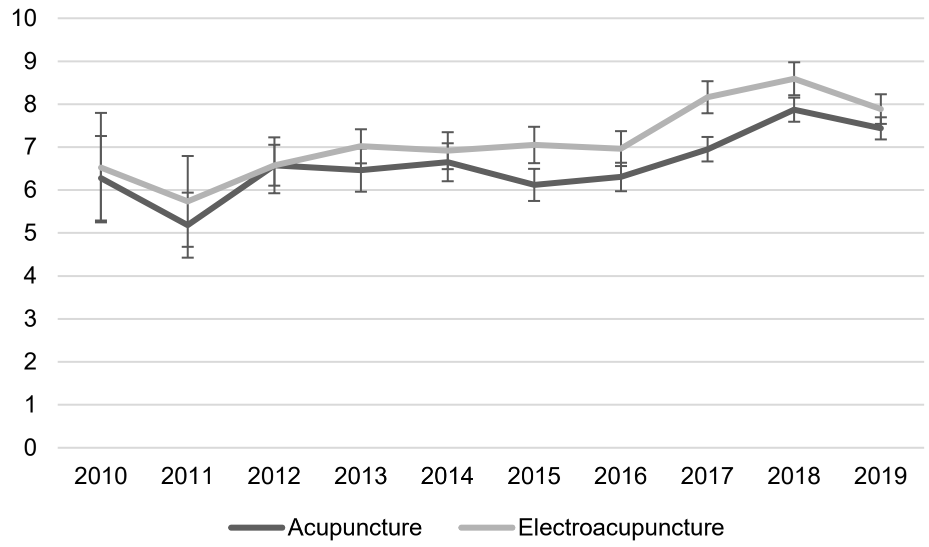

© 2024 Candon M et al. *JAMA Network Open.*

eTable 8. Logistic Regressions Predicting Any Acupuncture Use among Patients with Low Back Pain and Medicare Advantage, 2010-2019

	**Any Acupuncture**		**Any Electroacupuncture**	
	Odds ratio (99% CI)	p-value	Odds ratio (99% CI)	p-value
**Age (reference group: 75+ years)**				
18-24 years	-	-	-	-
25-34 years	0.47 (0.23-0.99)	0.008	0.38 (0.15-0.96)	0.007
35-44 years	0.50 (0.35-0.72)	<0.001	0.27 (0.14-0.53)	<0.001
45-54 years	0.51 (0.42-0.62)	<0.001	0.41 (0.32-0.53)	<0.001
55-64 years	0.60 (0.54-0.67)	<0.001	0.64 (0.55-0.74)	<0.001
65-74 years	1.30 (1.24-1.37)	<0.001	1.25 (1.18-1.33)	<0.001
**Race (reference group: White)**				
Asian	9.29 (8.79-9.81)	<0.001	18.3 (17.1-19.5)	<0.001
Black	0.61 (0.53-0.70)	<0.001	0.67 (0.55-0.82)	<0.001
Hispanic	1.06 (0.96-1.16)	0.087	1.32 (1.18-1.48)	<0.001
**Sex (reference group: Female)**				
Male	0.84 (0.80-0.89)	<0.001	0.99 (0.93-1.05)	0.695
**Clinical characteristics**				
Chronic low back pain	2.31 (2.22-2.41)	<0.001	2.30 (2.18-2.42)	<0.001
Any cancer	1.22 (1.16-1.30)	<0.001	1.26 (1.17-1.35)	<0.001
Any pregnancy	0.92 (0.44-1.92)	0.791	0.88 (0.33-2.37)	0.741
Charlson index	0.94 (0.93-0.95)	<0.001	0.92 (0.91-0.93)	<0.001
**Annual household income (reference group: Over $100,000)**				
Under $40,000	0.57 (0.53-0.62)	<0.001	0.64 (0.58-0.71)	<0.001
$40,000-$59,999	0.78 (0.72-0.84)	<0.001	0.78 (0.71-0.87)	<0.001
$60,000-$74,999	0.87 (0.80-0.95)	<0.001	0.89 (0.80-0.99)	0.004
$75,000-$99,999	0.87 (0.80-0.94)	<0.001	0.97 (0.88-1.07)	0.402
Missing	0.49 (0.43-0.57)	<0.001	0.44 (0.37-0.53)	<0.001
**Educational attainment (reference group: College)**				
High school or less	0.76 (0.70-0.83)	<0.001	1.03 (0.94-1.14)	0.341
Some college	0.74 (0.70-0.79)	<0.001	0.81 (0.74-0.87)	<0.001
Missing	0.72 (0.49-1.09)	0.037	0.69 (0.41-1.18)	0.075
**Region (reference group: Pacific)**				
East North Central	0.15 (0.13-0.16)	<0.001	0.11 (0.09-0.13)	<0.001
East South Central	0.03 (0.03-0.05)	<0.001	0.05 (0.03-0.09)	<0.001
Middle Atlantic	0.93 (0.87-0.98)	0.001	1.87 (1.75-2.00)	<0.001
Mountain	0.23 (0.21-0.26)	<0.001	0.19 (0.16-0.22)	<0.001

© 2024 Candon M et al. *JAMA Network Open.*

New England	0.47 (0.42-0.53)	<0.001	0.19 (0.16-0.22)	<0.001
South Atlantic	0.12 (0.11-0.13)	<0.001	0.10 (0.08-0.11)	<0.001
West North Central	0.28 (0.25-0.32)	<0.001	0.13 (0.11-0.16)	<0.001
West South Central	0.07 (0.06-0.09)	<0.001	0.07 (0.06-0.10)	<0.001
**Number of Patient-Years**	4,859,043		4,859,043	

© 2024 Candon M et al. *JAMA Network Open.*

eTable 9. Use of Nonpharmacologic, Pharmacologic, and Interventional Treatments among Patients with Low Back Pain and Medicare Advantage Disaggregated by Acupuncture/Electroacupuncture Use, 2010-2019

	**Users: Unadjusted mean**	**Non-users: Unadjusted mean**		**Users: Least-square mean**	**Non-users: Least-square mean**	
	share (99% CI)	share (99% CI)	p-value	share (99% CI)	share (99% CI)	p-value
**Nonpharmacologic Treatments**						
Chiropractic care	28.7% (28.1-29.3%)	12.5% (12.5-12.6%)	<0.001	23.2% (22.4-23.9%)	6.5% (6.0-6.9%)	<0.001
Occupational therapy	5.4% (4.9-5.8%)	6.3% (6.3-6.3%)	<0.001	4.5% (4.0-5.1%)	4.9% (4.6-5.3%)	0.217
Physical therapy	57.1% (56.3-58.0%)	36.1% (36.0-36.1%)	<0.001	51.8% (50.8-52.9%)	31.6% (30.9-32.2%)	<0.001
Psychotherapy	11.0% (10.6-11.5%)	7.4% (7.4-7.4%)	<0.001	22.5% (21.9-23.1%)	17.0% (16.7-17.4%)	<0.001
**Pharmacologic Treatments**						
Antidepressant	24.7% (23.9-25.6%)	37.7% (37.6-37.8%)	<0.001	41.1% (40.0-42.1%)	48.5% (47.9-49.2%)	<0.001
Gabapentinoid	25.0% (24.2-25.8%)	27.7% (27.6-27.8%)	<0.001	33.8% (32.8-34.7%)	34.3% (33.7-34.9%)	<0.001
Muscle relaxant	18.0% (17.2-18.8%)	28.0% (27.9-28.1%)	<0.001	35.6% (34.7-36.6%)	41.9% (41.3-42.5%)	<0.001
NSAID	42.2% (41.3-43.0%)	40.2% (40.1-40.3%)	<0.001	56.2% (55.1-57.2%)	55.3% (54.6-56.0%)	<0.001
Opioid	37.7% (36.8-38.6%)	57.3% (57.2-57.4%)	<0.001	51.2% (50.2-52.3%)	65.6% (64.9-66.2%)	<0.001
**Interventional Treatments**						
Epidural steroid injection	21.0% (20.3-21.6%)	15.6% (15.6-15.7%)	<0.001	19.6% (18.8-20.4%)	12.9% (12.4-13.4%)	<0.001
Facet joint interventions	10.6% (10.1-11.1%)	7.4% (7.4-7.4%)	<0.001	11.7% (11.1-12.3%)	7.5% (7.1-7.8%)	<0.001
Trigger point injection	10.8% (10.4-11.2%)	5.3% (5.3-5.4%)	<0.001	12.1% (11.6-12.6%)	6.0% (5.7-6.4%)	<0.001
Spinal cord stimulator	0.9% (0.8-1.1%)	0.7% (0.7-0.7%)	0.001	1.3% (1.1-1.5%)	0.9% (0.8-1.0%)	<0.001
**Number of Patients**	2,641,793			2,641,793		

© 2024 Candon M et al. *JAMA Network Open.*

eTable 10. Characteristics of Patients with Low Back Pain and Commercial Insurance, Stratified by Whether They Had 1+ Acupuncture or Electroacupuncture Claim, 2010-2019

	Used acupuncture	Used electroacupuncture	Never used acupuncture or electroacupuncture
	n (%)	n (%)	n (%)
**Age**	42.9 (SD=11.9)	43.5 (SD=11.7)	44.5 (SD=13.6)
**Race/ethnicity**			
Asian	17,068 (18.6)	17,230 (33.4)	155,884 (3.8)
Black	4,308 (4.7)	2,114 (4.1)	441,783 (10.8)
Hispanic	10,911 (11.9)	5,974 (11.6)	490,150 (12.0)
White	59,269 (64.7)	26,297 (51.0)	3,011,227 (73.5)
**Sex**			
Female	59,645 (65.2)	31,469 (61.0)	2,241,617 (54.7)
Male	31,911 (34.9)	20,146 (39.0)	1,857,427 (45.3)
**Clinical Characteristics**			
Chronic low back pain	54,070 (59.1)	31,551 (61.1)	1,212,611 (29.6)
Any cancer	9,843 (10.8)	5,184 (10.0)	327,315 (8.0)
Any pregnancy	9,309 (10.2)	4,393 (8.5)	149,247 (3.6)
**Annual household income**			
Under $40,000	9,732 (10.6)	4,851 (9.4)	752,521 (18.4)
$40,000-$59,999	7,508 (8.2)	3,883 (7.5)	540,717 (13.2)
$60,000-$74,999	6,942 (7.6)	3,702 (7.2)	395,041 (9.6)
$75,000-$99,999	11,315 (12.4)	5,711 (11.1)	605,963 (14.8)
Over $100,000	47,851 (52.3)	28,581 (55.4)	1,404,684 (34.3)
Missing	8,208 (9.0)	4,887 (9.5)	400,118 (9.8)
**Educational attainment**			
High school or less	7,663 (8.4)	4,144 (8.0)	1,024,109 (25.0)
Some college	40,677 (44.4)	20,843 (40.4)	2,245,325 (54.8)
College	42,724 (46.7)	26,419 (51.2)	804,863 (19.6)
Missing	492 (0.5)	209 (0.4)	24,747 (0.6)
**Region**			
East North Central	3,631 (4.0)	1,557 (3.0)	613,277 (15.0)
East South Central	415 (0.5)	173 (0.3)	212,008 (5.2)
Middle Atlantic	13,319 (14.6)	7,634 (14.8)	245,018 (6.0)
Mountain	7,907 (8.6)	2,969 (5.8)	398,469 (9.7)
New England	2,140 (2.3)	579 (1.1)	108,171 (2.6)
Pacific	39,106 (42.7)	28,390 (55.0)	371,496 (9.1)

© 2024 Candon M et al. *JAMA Network Open.*

South Atlantic	11,317 (12.4)	5,468 (10.6)	1,064,477 (26.0)
West North Central	7,244 (7.9)	1,809 (3.5)	458,907 (11.2)
West South Central	7,711 (8.4)	3,682 (7.1)	662,543 (16.2)
**Number of Patients**	91,556	51,615	4,099,044

© 2024 Candon M et al. *JAMA Network Open.*

eFigure 9. Share of Patients with Low Back Pain and Commercial Insurance with Any Acupuncture or Electroacupuncture Claim, 2010-2019

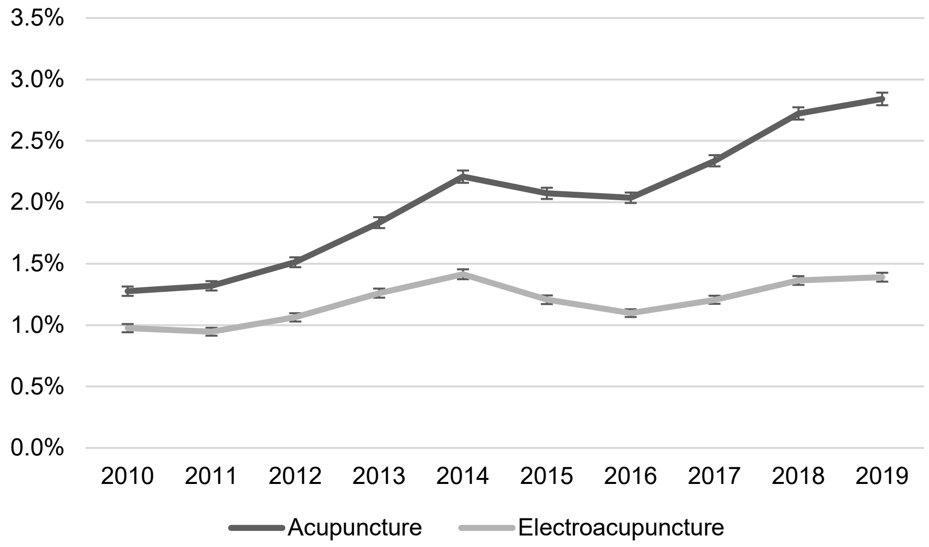

© 2024 Candon M et al. *JAMA Network Open.*

eFigure 10. Mean Number of Visits among Patients with Low Back Pain and Commercial Insurance with Any Acupuncture or Electroacupuncture Claim, 2010-2019

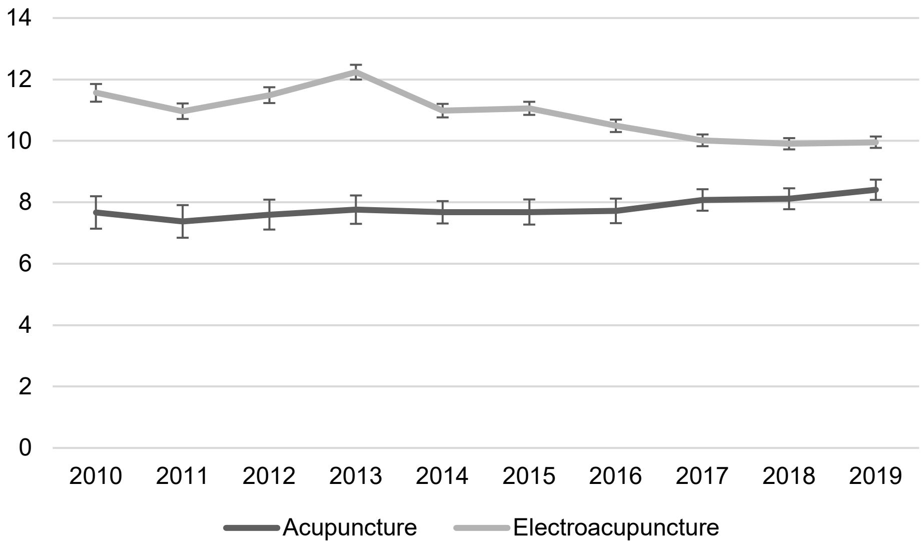

© 2024 Candon M et al. *JAMA Network Open.*

eTable 11. Logistic Regressions Predicting Any Acupuncture Use among Patients with Low Back Pain and Commercial Insurance, 2010-2019

	**Any Acupuncture**		**Any Electroacupuncture**	
	Odds ratio (99% CI)	p-value	Odds ratio (99% CI)	p-value
**Age (reference group: 75+ years)**				
18-24 years	1.79 (1.56-2.05)	<0.001	1.30 (1.08-1.56)	<0.001
25-34 years	3.18 (2.79-3.63)	<0.001	2.08 (1.74-2.47)	<0.001
35-44 years	3.38 (2.97-3.86)	<0.001	2.41 (2.02-2.86)	<0.001
45-54 years	2.81 (2.46-2.68)	<0.001	2.29 (1.93-2.72)	<0.001
55-64 years	2.35 (2.06-2.68)	<0.001	1.96 (1.65-2.33)	<0.001
65-74 years	2.12 (1.85-2.43)	<0.001	1.83 (1.53-2.19)	<0.001
**Race (reference group: White)**				
Asian	2.55 (2.48-2.63)	<0.001	6.23 (6.03-6.44)	<0.001
Black	0.96 (0.92-1.01)	0.042	1.14 (1.06-1.22)	<0.001
Hispanic	1.24 (1.20-1.29)	<0.001	1.47 (1.41-1.54)	<0.001
**Sex (reference group: Female)**				
Male	0.65 (0.64-0.67)	<0.001	0.79 (0.77-0.81)	<0.001
**Clinical characteristics**				
Chronic low back pain	2.50 (2.46-2.55)	<0.001	2.77 (2.71-2.84)	<0.001
Any cancer	1.26 (1.21-1.31)	<0.001	1.16 (1.10-1.22)	<0.001
Any pregnancy	1.86 (1.80-1.93)	<0.001	1.36 (1.29-1.43)	<0.001
Charlson index	0.97 (0.96-0.97)	<0.001	0.96 (0.94-0.97)	<0.001
**Annual household income (reference group: Over $100,000)**				
Under $40,000	0.78 (0.75-0.81)	<0.001	0.71 (0.67-0.74)	<0.001
$40,000-$59,999	0.82 (0.79-0.85)	<0.001	0.75 (0.71-0.80)	<0.001
$60,000-$74,999	0.90 (0.86-0.93)	<0.001	0.81 (0.77-0.86)	<0.001
$75,000-$99,999	0.90 (0.87-0.93)	<0.001	0.80 (0.76-0.84)	<0.001
Missing	0.84 (0.81-0.87)	<0.001	0.79 (0.75-0.83)	<0.001
**Educational attainment (reference group: College)**				
High school or less	0.28 (0.27-0.29)	<0.001	0.32 (0.30-0.33)	<0.001
Some college	0.51 (0.49-0.52)	<0.001	0.51 (0.50-0.53)	<0.001
Missing	0.70 (0.61-0.80)	<0.001	0.64 (0.52-0.79)	<0.001
**Region (reference group: Pacific)**				
East North Central	0.07 (0.07-0.08)	<0.001	0.05 (0.05-0.06)	<0.001
East South Central	0.03 (0.03-0.03)	<0.001	0.02 (0.02-0.03)	<0.001
Middle Atlantic	0.59 (0.57-0.60)	<0.001	0.48 (0.46-0.50)	<0.001
Mountain	0.23 (0.22-0.24)	<0.001	0.14 (0.13-0.15)	<0.001

© 2024 Candon M et al. *JAMA Network Open.*

New England	0.24 (0.22-0.25)	<0.001	0.10 (0.09-0.11)	<0.001
South Atlantic	0.14 (0.14-0.15)	<0.001	0.11 (0.10-0.11)	<0.001
West North Central	0.17 (0.17-0.18)	<0.001	0.07 (0.07-0.08)	<0.001
West South Central	0.16 (0.16-0.17)	<0.001	0.12 (0.11-0.13)	<0.001
**Number of Patient-Years**	6,379,361		6,379,361	

© 2024 Candon M et al. *JAMA Network Open.*

eTable 12. Use of Nonpharmacologic, Pharmacologic, and Interventional Treatments among Patients with Low Back Pain and Commercial Insurance Disaggregated by Acupuncture/Electroacupuncture Use, 2010-2019

	**Users: Unadjusted mean**	**Non-users: Unadjusted mean**		**Users: Least-square mean**	**Non-users: Least-square mean**	
	share (99% CI)	share (99% CI)	p-value	share (99% CI)	share (99% CI)	p-value
**Nonpharmacologic Treatments**						
Chiropractic care	47.8% (47.5-49.2%)	29.8% (29.7-29.9%)	<0.001	41.7% (41.4-42.0%)	23.5% (23.4-23.6%)	<0.001
Occupational therapy	3.1% (3.0-3.2%)	1.8% (1.8-1.8%)	<0.001	3.9% (3.8-4.0%)	2.4% (2.4-2.5%)	<0.001
Physical therapy	36.0% (35.6-36.3%)	24.9% (24.8-24.9%)	<0.001	38.6% (38.2-38.9%)	26.8% (26.6-26.9%)	<0.001
Psychotherapy	19.8% (19.6-20.0%)	8.9% (8.9-8.9%)	<0.001	17.1% (16.9-17.3%)	5.4% (5.4-5.5%)	<0.001
**Pharmacologic Treatments**						
Antidepressant	25.6% (25.3-25.9%)	26.3% (26.3-26.4%)	<0.001	19.9% (19.6-20.2%)	18.7% (18.6-18.8%)	<0.001
Gabapentinoid	11.9% (11.6-12.1%)	11.9% (11.9-12.0%)	0.470	11.0% (10.7-11.2%)	10.4% (10.3-10.4%)	<0.001
Muscle relaxant	25.4% (25.0-25.8%)	37.6% (37.5-37.6%)	<0.001	20.2% (19.9-20.6%)	31.6% (31.5-31.7%)	<0.001
NSAID	36.9% (36.5-37.2%)	42.3% (42.2-42.4%)	<0.001	36.9% (36.5-37.3%)	41.8% (41.7-42.0%)	<0.001
Opioid	42.0% (41.6-42.3%)	49.4% (49.3-49.5%)	<0.001	38.2% (37.8-38.6%)	43.5% (43.3-43.6%)	<0.001
**Interventional Treatments**						
Epidural steroid injection	9.5% (9.3-9.7%)	8.7% (8.6-8.7%)	<0.001	10.2% (10.0-10.4%)	8.6% (8.5-8.7%)	<0.001
Facet joint interventions	4.2% (4.0-4.3%)	3.2% (3.2-3.3%)	<0.001	4.1% (4.0-4.3%)	2.9% (2.9-2.9%)	<0.001
Trigger point injection	6.6% (6.5-6.8%)	3.4% (3.3-3.4%)	<0.001	6.2% (6.0-6.3%)	2.7% (2.7-2.8%)	<0.001
Spinal cord stimulator	0.3% (0.3-0.3%)	0.2% (0.2-0.2%)	<0.001	0.3% (0.2-0.3%)	0.2% (0.2-0.2%)	0.555
**Number of Patients**	4,217,022			4,217,022		

© 2024 Candon M et al. *JAMA Network Open.*

© 2024 Candon M et al. *JAMA Network Open*.
