# Supplementary material for: Utilization of Reimbursed Acupuncture Therapy for Low Back Pain
Source: JAMA Netw Open. 2024 Aug 29;7(8):e2430906. doi: 10.1001/jamanetworkopen.2024.30906 (PMC11362866; doi:10.1001/jamanetworkopen.2024.30906)
Supplement: Supplement 2. — Data Sharing Statement [file jamanetwopen-e2430906-s002.pdf]

Data Sharing Statement

Candon. Utilization of Reimbursed Acupuncture Therapy for Low Back Pain. *JAMA Netw Open*. Published August 29, 2024. doi:10.1001/jamanetworkopen.2024.30906

Data
Data available: No

Additional Information
Explanation for why data not available: We are happy to share our Stata code to generate results. However, the data are owned by Optum so users would need to secure a data license agreement to access.
